# 3-Methyl­benzo[1,2-*c*:5,4-*c*′]dichromen-6(8*H*)-one

**DOI:** 10.1107/S1600536814015001

**Published:** 2014-07-02

**Authors:** M. Kayalvizhi, G. Vasuki, Adil I. Khatri, Shriniwas D. Samant

**Affiliations:** a Department of Physics, Kunthavai Naachiar Government Arts College (W) (Autonomous), Thanjavur 613 007, Tamilnadu, India; b Department of Chemistry, Institute of Chemical Technology, N.M. Parekh Road, Matunga, Mumbai 400 019, Tamilnadu, India

**Keywords:** crystal structure

## Abstract

The title compound, C_21_H_14_O_3_, crystallizes with eight independent mol­ecules (*A*–*H*) in the asymmetric unit which are arranged in four groups of two mol­ecules each (*AB*, *CD*, *EF* and *GH*). In each mol­ecule, the pyran-2-one ring is planar (r.m.s. deviations vary from 0.001 to 0.017 Å), while the pyran ring has a screw-boat conformation. In the crystal, mol­ecules stack in two columns, along the [10-1] direction, composed of mol­ecules *C*, *B*, *E* and *G*, and *D*, *A*, *F* and *H*. Mol­ecules *A* and *F* are linked *via* C—H⋯O hydrogen bonds. In addition, there are a number of C—H⋯π contacts present involving all of the mol­ecules. These inter­actions result in the formation of a three-dimensional network.

## Related literature   

For the photosensitizing properties of coumarins, see: Kaidbey & Kligman (1981 ▶). For their medicinal applications, see: Kayalvizhi *et al.* (2013 ▶). For the synthesis of the title compound, see: Khatri & Samant (2014 ▶).
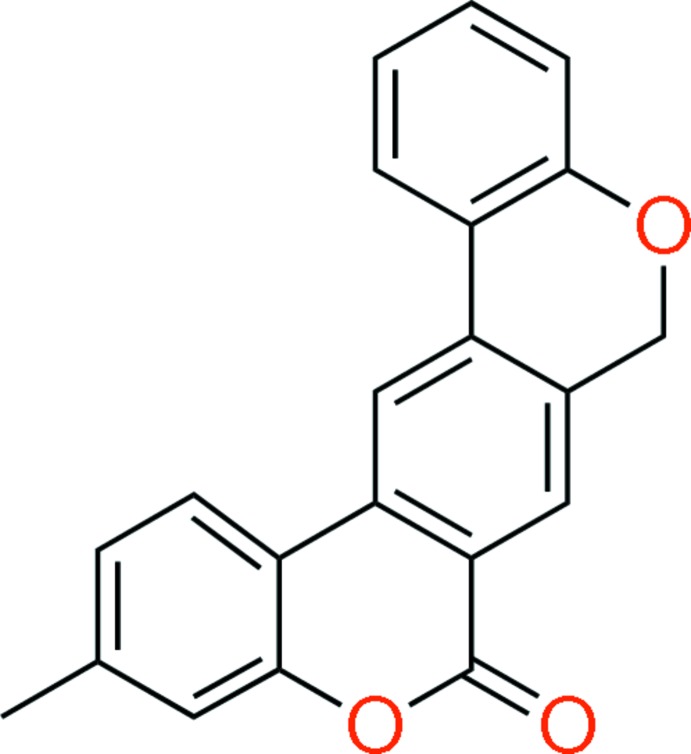


## Experimental   

### 

#### Crystal data  

C_21_H_14_O_3_
*M* *_r_* = 314.32
Monoclinic, 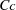

*a* = 20.7595 (16) Å
*b* = 20.7800 (16) Å
*c* = 28.427 (2) Å
β = 100.489 (2)°
*V* = 12058.1 (16) Å^3^
*Z* = 32
Mo *K*α radiation
μ = 0.09 mm^−1^
*T* = 296 K
0.40 × 0.35 × 0.30 mm

#### Data collection  

Bruker Kappa APEXII CCD diffractometer
Absorption correction: multi-scan (*SADABS*; Sheldrick, 1996 ▶) *T*_min_ = 0.972, *T*_max_ = 1.000
55067 measured reflections
22280 independent reflections
11040 reflections with *I* > 2σ(*I*)
*R*_int_ = 0.054

#### Refinement  

*R*[*F* ^2^ > 2σ(*F* ^2^)] = 0.083
*wR*(*F* ^2^) = 0.289
*S* = 1.07
22280 reflections
1737 parameters
2 restraints
H-atom parameters constrained
Δρ_max_ = 0.57 e Å^−3^
Δρ_min_ = −0.32 e Å^−3^

### 

Data collection: *APEX2* (Bruker, 2004 ▶); cell refinement: *APEX2* and *SAINT* (Bruker, 2004 ▶); data reduction: *SAINT* and *XPREP* (Bruker, 2004 ▶); program(s) used to solve structure: *SHELXS97* (Sheldrick, 2008 ▶); program(s) used to refine structure: *SHELXL97* (Sheldrick, 2008 ▶); molecular graphics: *ORTEP-3 for Windows* (Farrugia, 2012 ▶); software used to prepare material for publication: *PLATON* (Spek, 2009 ▶).

## Supplementary Material

Crystal structure: contains datablock(s) I, global. DOI: 10.1107/S1600536814015001/su2746sup1.cif

Structure factors: contains datablock(s) I. DOI: 10.1107/S1600536814015001/su2746Isup2.hkl

Supporting information file. DOI: 10.1107/S1600536814015001/su2746Isup3.cml

CCDC reference: 1010326

Additional supporting information:  crystallographic information; 3D view; checkCIF report

## Figures and Tables

**Table 1:** Hydrogen-bond geometry (Å, °) *Cg*24, *Cg*9, *Cg*39, *Cg*34, *Cg*4 and *Cg*14 are the centroids of rings C6*E*–C11*E*, C6*B*–C11*B*, C6*H*–C11*H*, C6*G*–C11*G*, C6*A*–C11*A* and C6*C*–C11*C*, respectively.

*D*—H⋯*A*	*D*—H	H⋯*A*	*D*⋯*A*	*D*—H⋯*A*
C7*A*—H7*A*⋯O3*F* ^i^	0.93	2.64	3.520 (13)	157
C7*F*—H7*F*⋯O3*A* ^ii^	0.93	2.64	3.526 (12)	159
C21*B*—H21*E*⋯*Cg*24	0.96	2.68	3.575 (13)	155
C21*C*—H21*H*⋯*Cg*9^ii^	0.96	2.80	3.554 (12)	136
C21*D*—H21*K*⋯*Cg*39^iii^	0.96	2.66	3.553 (12)	154
C21*E*—H21*M*⋯*Cg*34	0.96	2.92	3.622 (13)	131
C21*F*—H21*Q*⋯*Cg*4^i^	0.96	2.68	3.575 (13)	156
C21*G*—H21*S*⋯*Cg*14^iii^	0.96	2.66	3.556 (12)	155

Symmetry codes: (i) 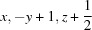; (ii) 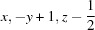; (iii) 
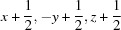
.

**Fig. 1.**
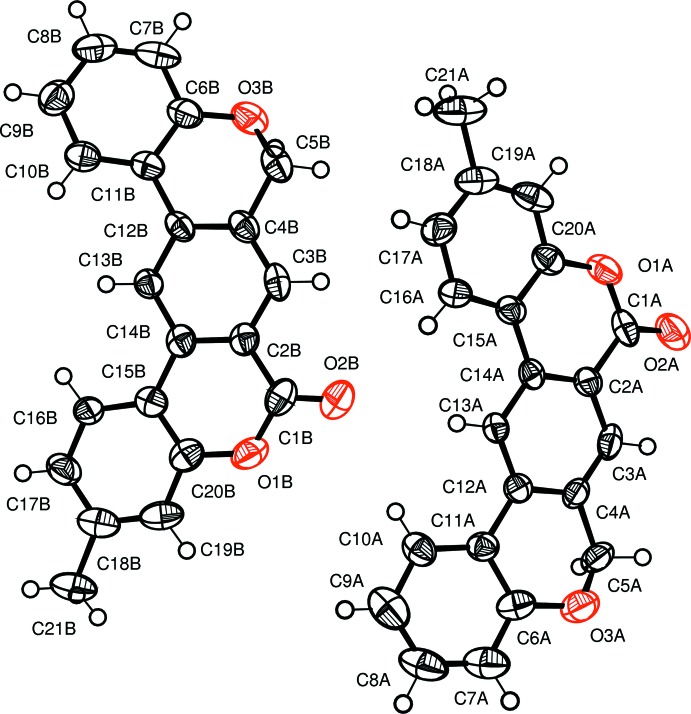
The molecular structure of molecules A and B of the title compound, with atom labelling. The displacement ellipsoids are drawn at the 50% probability level.

**Fig. 2.**
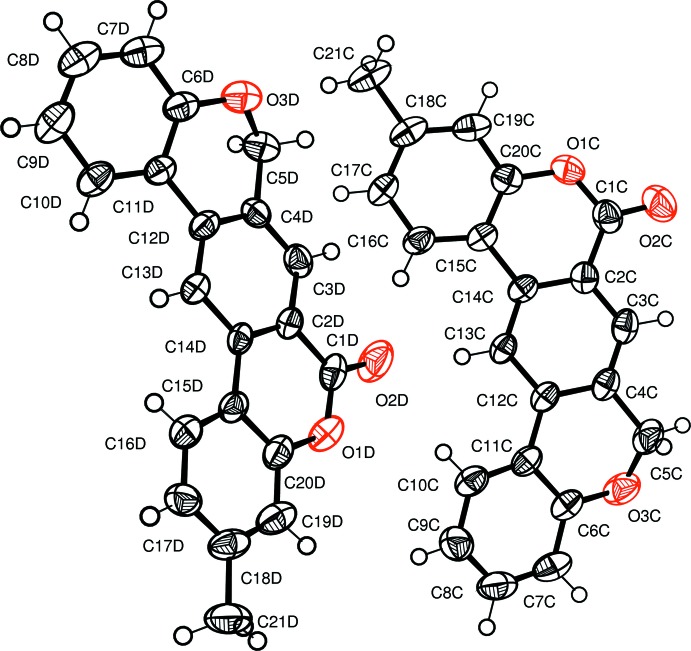
The molecular structure of molecules C and D of the title compound, with atom labelling. The displacement ellipsoids are drawn at the 50% probability level.

**Fig. 3.**
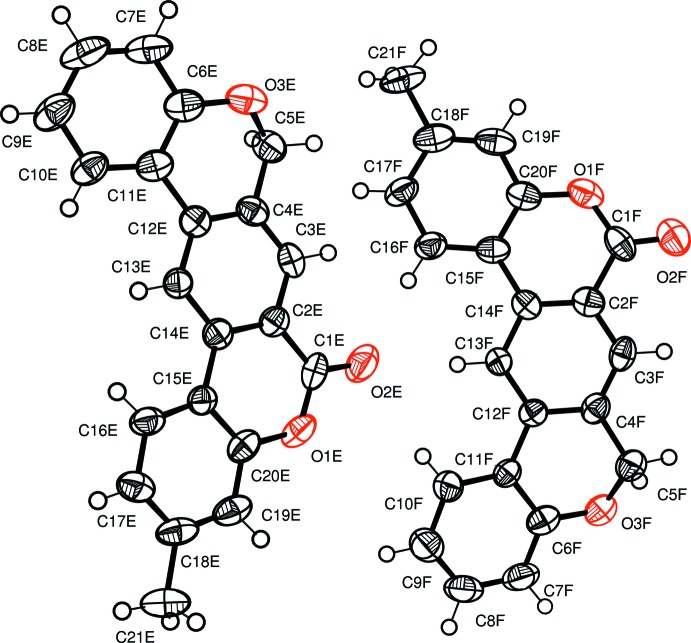
The molecular structure of molecules E and F of the title compound, with atom labelling. The displacement ellipsoids are drawn at the 50% probability level.

**Fig. 4.**
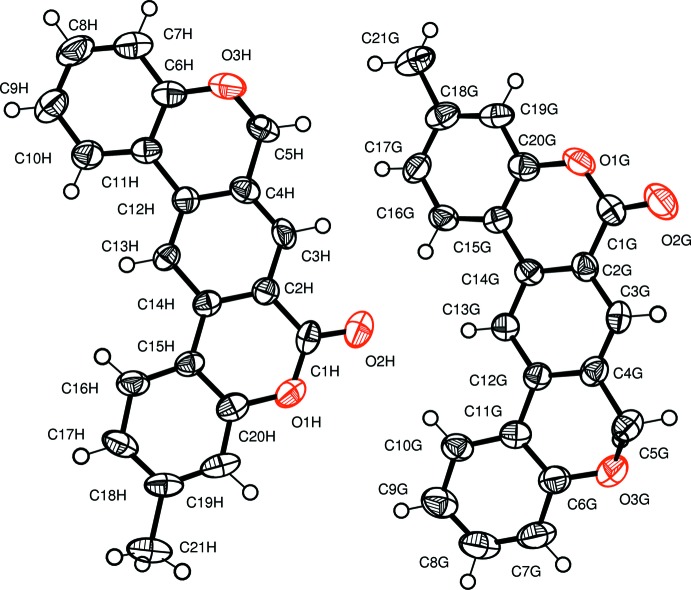
The molecular structure of molecules G and H of the title compound, with atom labelling. The displacement ellipsoids are drawn at the 50% probability level.

**Fig. 5.**
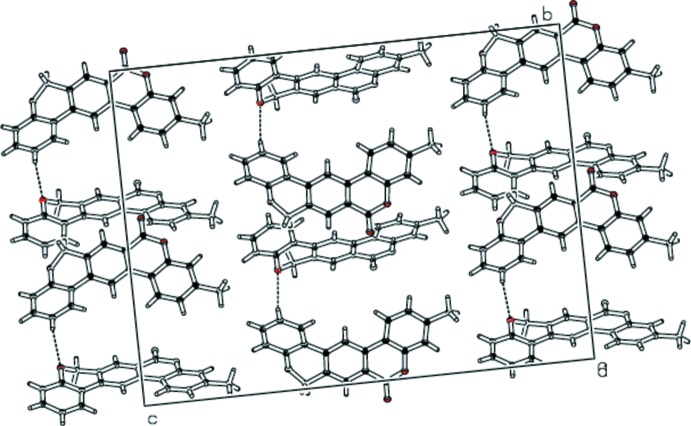
Crystal packing of the title compound viewed along the b axis, showing the C—H···O hydrogen bonds as dashed lines (see Table 1 for details).

**Fig. 6.**
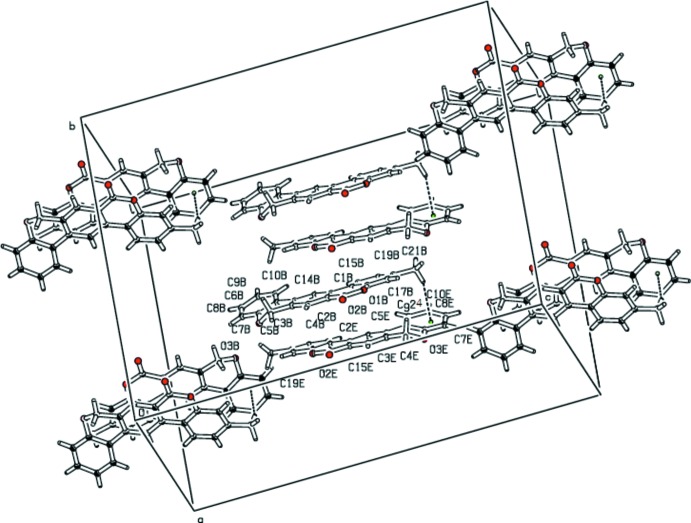
Crystal packing of the title compound viewed along the b axis, showing the C—H···π interactions as dashed lines (see Table 1 for details).

## supplementary crystallographic information

### S1. Comment

Coumarin is the simplest member of the group of oxygen heterocyclic compounds called benzo-2-pyrones. Coumarins are an important class of compound due to their presence in natural products as well as their medicinal applications, *e.g.* as anti-inflammatory, anti-viral, antioxidant, antibacterial, antifungal, anti-HIV and as anti-carcinogenic agents. Coumarin and its derivatives also have applications as fluorescent dyes for synthetic fibres and daylight fluorescent pigments (Kayalvizhi *et al.*., 2013). Coumarin and several of its derivatives were investigated for their photosensitizing properties. With a few exceptions, the coumarins are potentially strong photocontact sensitizers but do not evoke phototoxic reactions (Kaidbey & Kligman, 1981).

The title compound, crystallizes with eight independent molecules in the asymmetric unit, which have been labelled in an identical manner (Fig. 1, 2, 3 and 4) and are distinguished by suffixes A, B, C, D, E, F, G and H, respectively. The asymmetric unit is composed of four groups of two molecules each (AB, CD, EF & GH). In each groups the molecules differ in their orientation. In each molecule, the pyran-2-one ring is planar, while the pyran ring has a screw-boat conformation.

In the crystal, molecules A and F are linked *via* C—H···O hydrogen bonds; between atoms C7A and O3F^i^ and C7F and O3A^ii^ (Table 1 and Fig. 5). In addition, a number of C—H···π interactions are observed (Table 1 and Fig. 6).

### S2. Experimental

The compound was synthesized according to the published procedure (Khatri & Samant, 2014).

### S3. Refinement

All the H atoms were positioned geometrically and treated as riding on their parent atoms: C—H = 0.93 Å (aromatic), 0.96 Å (methyl) and 0.97 Å (methylene) with *U*_iso_(H) = 1.5*U*_eq_(C-methyl) and = 1.2*U*_eq_(C) for other H atoms.

### Crystal data

**Table:**

C_21_H_14_O_3_	*F*(000) = 5248
*M**_r_* = 314.32	*D*_x_ = 1.385 Mg m^−^^3^
Monoclinic, *C**c*	Mo *K*α radiation, λ = 0.71073 Å
*a* = 20.7595 (16) Å	Cell parameters from 22280 reflections
*b* = 20.7800 (16) Å	θ = 1.4–26.4°
*c* = 28.427 (2) Å	µ = 0.09 mm^−^^1^
β = 100.489 (2)°	*T* = 296 K
*V* = 12058.1 (16) Å^3^	Block, colourless
*Z* = 32	0.40 × 0.35 × 0.30 mm

### Data collection

**Table:**

Bruker Kappa APEXII CCD diffractometer	22280 independent reflections
Radiation source: fine-focus sealed tube	11040 reflections with *I* > 2σ(*I*)
Graphite monochromator	*R*_int_ = 0.054
ω and φ scan	θ_max_ = 26.4°, θ_min_ = 1.4°
Absorption correction: multi-scan (*SADABS*; Sheldrick, 1996)	*h* = −25→21
*T*_min_ = 0.972, *T*_max_ = 1.000	*k* = −16→25
55067 measured reflections	*l* = −35→35

### Refinement

**Table:**

Refinement on *F*^2^	Primary atom site location: structure-invariant direct methods
Least-squares matrix: full	Secondary atom site location: difference Fourier map
*R*[*F*^2^ > 2σ(*F*^2^)] = 0.083	Hydrogen site location: inferred from neighbouring sites
*wR*(*F*^2^) = 0.289	H-atom parameters constrained
*S* = 1.07	*w* = 1/[σ^2^(*F*_o_^2^) + (0.1593*P*)^2^] where *P* = (*F*_o_^2^ + 2*F*_c_^2^)/3
22280 reflections	(Δ/σ)_max_ = 0.021
1737 parameters	Δρ_max_ = 0.57 e Å^−^^3^
2 restraints	Δρ_min_ = −0.32 e Å^−^^3^

### Special details

**Table:**

Geometry. All e.s.d.'s (except the e.s.d. in the dihedral angle between two l.s. planes) are estimated using the full covariance matrix. The cell e.s.d.'s are taken into account individually in the estimation of e.s.d.'s in distances, angles and torsion angles; correlations between e.s.d.'s in cell parameters are only used when they are defined by crystal symmetry. An approximate (isotropic) treatment of cell e.s.d.'s is used for estimating e.s.d.'s involving l.s. planes.

### Fractional atomic coordinates and isotropic or equivalent isotropic displacement parameters (Å^2^)

**Table:**

	*x*	*y*	*z*	*U*_iso_*/*U*_eq_	
C1A	0.3430 (4)	0.4971 (5)	0.4635 (4)	0.047 (2)	
C1B	0.1154 (5)	0.2412 (4)	0.5303 (4)	0.048 (3)	
C1C	−0.0282 (5)	0.3664 (5)	0.4641 (4)	0.046 (2)	
C1D	0.2348 (5)	0.6209 (4)	0.5316 (4)	0.044 (2)	
C1E	0.2231 (5)	0.1163 (4)	0.4641 (4)	0.045 (2)	
C1F	0.4837 (5)	0.3711 (5)	0.5309 (4)	0.048 (2)	
C1G	0.3631 (5)	−0.0099 (4)	0.5297 (4)	0.043 (2)	
C1H	0.5916 (4)	0.2446 (4)	0.4629 (4)	0.043 (2)	
C2A	0.3714 (4)	0.5352 (4)	0.5062 (3)	0.0354 (19)	
C2B	0.1422 (4)	0.2596 (4)	0.4881 (3)	0.037 (2)	
C2C	0.0197 (4)	0.3834 (4)	0.5065 (3)	0.039 (2)	
C2D	0.2408 (4)	0.6575 (4)	0.4887 (3)	0.037 (2)	
C2E	0.2707 (4)	0.1333 (4)	0.5064 (3)	0.037 (2)	
C2F	0.4914 (4)	0.4092 (4)	0.4884 (3)	0.040 (2)	
C2G	0.3887 (4)	0.0079 (4)	0.4875 (3)	0.038 (2)	
C2H	0.6191 (4)	0.2817 (4)	0.5054 (3)	0.0357 (19)	
C3A	0.3678 (4)	0.5101 (4)	0.5515 (4)	0.043 (2)	
H3A	0.3466	0.4711	0.5539	0.052*	
C3B	0.1045 (4)	0.2440 (4)	0.4433 (4)	0.044 (2)	
H3B	0.0651	0.2218	0.4411	0.053*	
C3C	0.0060 (5)	0.3694 (4)	0.5518 (4)	0.045 (2)	
H3C	−0.0326	0.3480	0.5542	0.054*	
C3D	0.2145 (4)	0.6311 (4)	0.4443 (3)	0.040 (2)	
H3D	0.1927	0.5918	0.4422	0.048*	
C3E	0.2561 (4)	0.1189 (4)	0.5517 (4)	0.041 (2)	
H3E	0.2176	0.0973	0.5541	0.049*	
C3F	0.4652 (4)	0.3846 (4)	0.4433 (4)	0.042 (2)	
H3F	0.4425	0.3458	0.4407	0.050*	
C3G	0.3520 (4)	−0.0071 (4)	0.4422 (4)	0.040 (2)	
H3G	0.3126	−0.0291	0.4398	0.048*	
C3H	0.6160 (4)	0.2562 (4)	0.5508 (3)	0.042 (2)	
H3H	0.5951	0.2171	0.5533	0.050*	
C4A	0.3951 (4)	0.5423 (4)	0.5917 (3)	0.0363 (19)	
C4B	0.1272 (4)	0.2625 (4)	0.4026 (3)	0.039 (2)	
C4C	0.0483 (5)	0.3863 (4)	0.5924 (3)	0.040 (2)	
C4D	0.2213 (4)	0.6643 (4)	0.4031 (3)	0.037 (2)	
C4E	0.2987 (4)	0.1367 (4)	0.5927 (3)	0.038 (2)	
C4F	0.4727 (4)	0.4171 (4)	0.4029 (3)	0.039 (2)	
C4G	0.3744 (5)	0.0109 (4)	0.4014 (3)	0.041 (2)	
C4H	0.6434 (4)	0.2884 (4)	0.5910 (3)	0.040 (2)	
C5A	0.3937 (5)	0.5153 (5)	0.6404 (3)	0.046 (2)	
H5A	0.3565	0.4866	0.6385	0.056*	
H5B	0.4332	0.4903	0.6510	0.056*	
C5B	0.0870 (5)	0.2493 (5)	0.3539 (4)	0.050 (2)	
H5C	0.0592	0.2123	0.3559	0.060*	
H5D	0.0589	0.2860	0.3440	0.060*	
C5C	0.0338 (5)	0.3745 (5)	0.6409 (4)	0.054 (3)	
H5E	0.0118	0.4120	0.6508	0.064*	
H5F	0.0040	0.3383	0.6394	0.064*	
C5D	0.1963 (5)	0.6379 (5)	0.3545 (3)	0.052 (2)	
H5G	0.2310	0.6134	0.3441	0.062*	
H5H	0.1605	0.6086	0.3563	0.062*	
C5E	0.2835 (5)	0.1232 (5)	0.6409 (3)	0.050 (2)	
H5I	0.2603	0.1596	0.6511	0.060*	
H5J	0.2549	0.0860	0.6389	0.060*	
C5F	0.4462 (5)	0.3914 (5)	0.3537 (3)	0.049 (2)	
H5K	0.4799	0.3661	0.3428	0.058*	
H5L	0.4095	0.3631	0.3553	0.058*	
C5G	0.3351 (5)	−0.0012 (5)	0.3533 (3)	0.050 (2)	
H5M	0.3066	−0.0378	0.3549	0.060*	
H5N	0.3078	0.0360	0.3434	0.060*	
C5H	0.6422 (5)	0.2617 (5)	0.6399 (3)	0.050 (2)	
H5O	0.6049	0.2331	0.6381	0.060*	
H5P	0.6816	0.2365	0.6503	0.060*	
C6A	0.4354 (5)	0.6132 (4)	0.6762 (3)	0.047 (2)	
C6B	0.1756 (5)	0.2829 (4)	0.3178 (3)	0.046 (2)	
C6C	0.1392 (5)	0.4070 (4)	0.6764 (3)	0.044 (2)	
C6D	0.2183 (4)	0.7341 (4)	0.3182 (3)	0.041 (2)	
C6E	0.3892 (5)	0.1555 (5)	0.6766 (3)	0.048 (2)	
C6F	0.4715 (5)	0.4886 (4)	0.3187 (3)	0.045 (2)	
C6G	0.4231 (5)	0.0301 (5)	0.3173 (3)	0.047 (2)	
C6H	0.6838 (5)	0.3586 (5)	0.6760 (3)	0.044 (2)	
C7A	0.4576 (6)	0.6437 (5)	0.7195 (4)	0.063 (3)	
H7A	0.4416	0.6315	0.7466	0.075*	
C7B	0.1948 (6)	0.2965 (6)	0.2743 (3)	0.064 (3)	
H7B	0.1746	0.2754	0.2467	0.077*	
C7C	0.1798 (5)	0.4196 (5)	0.7201 (3)	0.057 (3)	
H7C	0.1728	0.3985	0.7476	0.069*	
C7D	0.2196 (5)	0.7634 (5)	0.2747 (3)	0.054 (3)	
H7D	0.1919	0.7492	0.2472	0.065*	
C7E	0.4308 (5)	0.1677 (6)	0.7206 (4)	0.062 (3)	
H7E	0.4248	0.1459	0.7481	0.075*	
C7F	0.4726 (6)	0.5193 (5)	0.2756 (4)	0.060 (3)	
H7F	0.4437	0.5066	0.2483	0.072*	
C7G	0.4429 (6)	0.0420 (6)	0.2735 (4)	0.065 (3)	
H7G	0.4234	0.0201	0.2460	0.078*	
C7H	0.7070 (5)	0.3888 (5)	0.7191 (4)	0.057 (3)	
H7H	0.6928	0.3749	0.7466	0.068*	
C8A	0.5024 (6)	0.6909 (5)	0.7222 (4)	0.063 (3)	
H8A	0.5182	0.7104	0.7514	0.075*	
C8B	0.2425 (5)	0.3400 (6)	0.2720 (4)	0.059 (3)	
H8B	0.2549	0.3484	0.2428	0.071*	
C8C	0.2301 (6)	0.4628 (5)	0.7228 (4)	0.059 (3)	
H8C	0.2577	0.4704	0.7519	0.071*	
C8D	0.2619 (6)	0.8131 (5)	0.2723 (4)	0.060 (3)	
H8D	0.2620	0.8333	0.2431	0.072*	
C8E	0.4794 (5)	0.2113 (6)	0.7228 (4)	0.067 (3)	
H8E	0.5068	0.2192	0.7519	0.081*	
C8F	0.5152 (6)	0.5675 (5)	0.2731 (4)	0.063 (3)	
H8F	0.5157	0.5877	0.2439	0.075*	
C8G	0.4910 (5)	0.0861 (6)	0.2715 (4)	0.064 (3)	
H8G	0.5036	0.0945	0.2424	0.077*	
C8H	0.7498 (6)	0.4377 (5)	0.7219 (4)	0.063 (3)	
H8H	0.7645	0.4579	0.7511	0.076*	
C9A	0.5251 (6)	0.7106 (5)	0.6822 (4)	0.063 (3)	
H9A	0.5564	0.7430	0.6845	0.076*	
C9B	0.2727 (5)	0.3717 (5)	0.3122 (4)	0.054 (3)	
H9B	0.3046	0.4025	0.3101	0.065*	
C9C	0.2395 (5)	0.4950 (5)	0.6822 (4)	0.057 (3)	
H9C	0.2721	0.5261	0.6840	0.068*	
C9D	0.3045 (6)	0.8340 (5)	0.3123 (4)	0.059 (3)	
H9D	0.3336	0.8673	0.3101	0.070*	
C9E	0.4892 (5)	0.2445 (6)	0.6823 (4)	0.060 (3)	
H9E	0.5222	0.2752	0.6846	0.072*	
C9F	0.5578 (6)	0.5869 (5)	0.3132 (4)	0.061 (3)	
H9F	0.5877	0.6197	0.3111	0.073*	
C9G	0.5213 (5)	0.1185 (5)	0.3115 (4)	0.058 (3)	
H9G	0.5533	0.1491	0.3094	0.069*	
C9H	0.7720 (6)	0.4581 (5)	0.6811 (4)	0.058 (3)	
H9H	0.8027	0.4910	0.6829	0.070*	
C10A	0.5011 (5)	0.6818 (4)	0.6376 (3)	0.044 (2)	
H10A	0.5161	0.6959	0.6105	0.053*	
C10B	0.2556 (5)	0.3577 (4)	0.3559 (3)	0.045 (2)	
H10B	0.2773	0.3786	0.3832	0.053*	
C10C	0.1997 (5)	0.4806 (4)	0.6385 (3)	0.045 (2)	
H10C	0.2072	0.5017	0.6111	0.054*	
C10D	0.3033 (5)	0.8040 (4)	0.3569 (3)	0.044 (2)	
H10D	0.3323	0.8171	0.3841	0.053*	
C10E	0.4497 (4)	0.2319 (5)	0.6382 (3)	0.046 (2)	
H10E	0.4567	0.2538	0.6111	0.056*	
C10F	0.5564 (5)	0.5572 (4)	0.3577 (4)	0.048 (2)	
H10F	0.5856	0.5703	0.3848	0.058*	
C10G	0.5034 (4)	0.1048 (4)	0.3554 (3)	0.045 (2)	
H10G	0.5248	0.1256	0.3828	0.054*	
C10H	0.7479 (5)	0.4285 (4)	0.6373 (3)	0.047 (2)	
H10H	0.7624	0.4425	0.6100	0.056*	
C11A	0.4559 (4)	0.6332 (4)	0.6338 (3)	0.037 (2)	
C11B	0.2067 (4)	0.3133 (4)	0.3599 (3)	0.037 (2)	
C11C	0.1496 (4)	0.4363 (4)	0.6345 (3)	0.038 (2)	
C11D	0.2586 (4)	0.7546 (4)	0.3599 (3)	0.0331 (18)	
C11E	0.4001 (4)	0.1866 (4)	0.6347 (3)	0.042 (2)	
C11F	0.5117 (4)	0.5086 (4)	0.3610 (3)	0.0338 (18)	
C11G	0.4539 (4)	0.0601 (4)	0.3591 (3)	0.039 (2)	
C11H	0.7028 (4)	0.3790 (4)	0.6336 (3)	0.0362 (19)	
C12A	0.4269 (4)	0.6012 (4)	0.5890 (3)	0.0338 (18)	
C12B	0.1864 (4)	0.2940 (4)	0.4050 (3)	0.0353 (19)	
C12C	0.1061 (4)	0.4187 (4)	0.5897 (3)	0.0339 (19)	
C12D	0.2531 (4)	0.7233 (4)	0.4055 (3)	0.0343 (19)	
C12E	0.3571 (4)	0.1686 (4)	0.5895 (3)	0.036 (2)	
C12F	0.5051 (4)	0.4763 (4)	0.4061 (3)	0.0335 (18)	
C12G	0.4338 (4)	0.0428 (4)	0.4043 (3)	0.0364 (19)	
C12H	0.6741 (4)	0.3478 (4)	0.5887 (3)	0.0340 (18)	
C13A	0.4297 (4)	0.6271 (4)	0.5440 (3)	0.0315 (18)	
H13A	0.4504	0.6664	0.5417	0.038*	
C13B	0.2239 (4)	0.3077 (4)	0.4499 (3)	0.0346 (19)	
H13B	0.2640	0.3286	0.4520	0.041*	
C13C	0.1214 (4)	0.4317 (4)	0.5445 (3)	0.0339 (19)	
H13C	0.1606	0.4521	0.5423	0.041*	
C13D	0.2788 (4)	0.7497 (4)	0.4497 (3)	0.0329 (18)	
H13D	0.3009	0.7889	0.4515	0.040*	
C13E	0.3716 (4)	0.1824 (4)	0.5446 (3)	0.0343 (19)	
H13E	0.4103	0.2037	0.5424	0.041*	
C13F	0.5306 (4)	0.5019 (4)	0.4507 (3)	0.0359 (19)	
H13F	0.5526	0.5410	0.4530	0.043*	
C13G	0.4715 (4)	0.0560 (4)	0.4490 (3)	0.0353 (19)	
H13G	0.5116	0.0768	0.4512	0.042*	
C13H	0.6768 (4)	0.3743 (4)	0.5438 (3)	0.0346 (19)	
H13H	0.6972	0.4138	0.5417	0.041*	
C14A	0.4012 (4)	0.5940 (4)	0.5023 (3)	0.0311 (18)	
C14B	0.2017 (4)	0.2901 (4)	0.4921 (3)	0.038 (2)	
C14C	0.0783 (4)	0.4143 (4)	0.5027 (3)	0.0353 (19)	
C14D	0.2715 (4)	0.7170 (4)	0.4923 (3)	0.0307 (17)	
C14E	0.3288 (4)	0.1647 (4)	0.5026 (3)	0.037 (2)	
C14F	0.5230 (4)	0.4683 (4)	0.4928 (3)	0.0359 (19)	
C14G	0.4491 (4)	0.0379 (4)	0.4910 (3)	0.0345 (19)	
C14H	0.6485 (4)	0.3413 (4)	0.5017 (3)	0.0340 (18)	
C15A	0.4029 (4)	0.6188 (4)	0.4548 (3)	0.0348 (19)	
C15B	0.2380 (4)	0.3034 (4)	0.5399 (3)	0.037 (2)	
C15C	0.0909 (4)	0.4278 (4)	0.4550 (3)	0.0354 (19)	
C15D	0.2976 (4)	0.7421 (4)	0.5398 (3)	0.0354 (19)	
C15E	0.3407 (4)	0.1784 (4)	0.4547 (3)	0.0336 (19)	
C15F	0.5485 (4)	0.4923 (4)	0.5403 (3)	0.039 (2)	
C15G	0.4853 (4)	0.0516 (4)	0.5387 (3)	0.0351 (19)	
C15H	0.6504 (4)	0.3661 (4)	0.4543 (3)	0.0355 (19)	
C16A	0.4288 (4)	0.6780 (4)	0.4445 (3)	0.041 (2)	
H16A	0.4474	0.7047	0.4696	0.049*	
C16B	0.2998 (4)	0.3324 (4)	0.5496 (3)	0.040 (2)	
H16B	0.3197	0.3449	0.5243	0.048*	
C16C	0.1481 (5)	0.4565 (4)	0.4452 (3)	0.043 (2)	
H16C	0.1808	0.4688	0.4705	0.051*	
C16D	0.3277 (4)	0.8012 (4)	0.5494 (3)	0.044 (2)	
H16D	0.3331	0.8276	0.5240	0.053*	
C16E	0.3972 (5)	0.2070 (4)	0.4451 (3)	0.043 (2)	
H16E	0.4299	0.2194	0.4703	0.051*	
C16F	0.5796 (4)	0.5522 (5)	0.5505 (3)	0.044 (2)	
H16F	0.5854	0.5789	0.5253	0.053*	
C16G	0.5473 (5)	0.0799 (4)	0.5487 (3)	0.044 (2)	
H16G	0.5673	0.0917	0.5233	0.053*	
C16H	0.6762 (4)	0.4246 (5)	0.4445 (3)	0.044 (2)	
H16H	0.6948	0.4512	0.4696	0.053*	
C17A	0.4277 (5)	0.6980 (5)	0.3979 (3)	0.047 (2)	
H17A	0.4453	0.7379	0.3925	0.056*	
C17B	0.3318 (5)	0.3427 (5)	0.5959 (3)	0.050 (3)	
H17B	0.3734	0.3609	0.6010	0.060*	
C17C	0.1571 (5)	0.4669 (4)	0.3991 (3)	0.045 (2)	
H17C	0.1962	0.4849	0.3938	0.053*	
C17D	0.3500 (5)	0.8221 (5)	0.5957 (3)	0.047 (2)	
H17D	0.3693	0.8625	0.6009	0.057*	
C17E	0.4055 (5)	0.2174 (5)	0.3984 (3)	0.049 (2)	
H17E	0.4444	0.2358	0.3931	0.059*	
C17F	0.6020 (5)	0.5727 (5)	0.5969 (3)	0.048 (2)	
H17F	0.6224	0.6125	0.6025	0.057*	
C17G	0.5800 (5)	0.0908 (4)	0.5948 (3)	0.048 (2)	
H17G	0.6215	0.1091	0.5999	0.057*	
C17H	0.6751 (5)	0.4446 (5)	0.3972 (3)	0.048 (2)	
H17H	0.6930	0.4843	0.3917	0.058*	
C18A	0.4012 (5)	0.6606 (5)	0.3595 (3)	0.049 (2)	
C18B	0.3038 (5)	0.3268 (5)	0.6349 (3)	0.051 (3)	
C18C	0.1085 (5)	0.4508 (4)	0.3601 (3)	0.048 (2)	
C18D	0.3437 (5)	0.7831 (5)	0.6349 (3)	0.047 (2)	
C18E	0.3588 (5)	0.2015 (5)	0.3602 (3)	0.048 (2)	
C18F	0.5939 (5)	0.5336 (5)	0.6359 (3)	0.050 (2)	
C18G	0.5506 (5)	0.0746 (4)	0.6336 (3)	0.050 (2)	
C18H	0.6482 (4)	0.4071 (5)	0.3590 (3)	0.045 (2)	
C19A	0.3742 (5)	0.6017 (5)	0.3687 (3)	0.051 (3)	
H19A	0.3555	0.5754	0.3434	0.061*	
C19B	0.2421 (5)	0.2978 (5)	0.6259 (3)	0.052 (3)	
H19B	0.2218	0.2862	0.6512	0.063*	
C19C	0.0510 (5)	0.4220 (4)	0.3691 (3)	0.049 (2)	
H19C	0.0179	0.4107	0.3438	0.059*	
C19D	0.3126 (5)	0.7250 (5)	0.6258 (3)	0.050 (2)	
H19D	0.3066	0.6987	0.6511	0.060*	
C19E	0.3023 (5)	0.1722 (4)	0.3688 (3)	0.048 (2)	
H19E	0.2695	0.1605	0.3434	0.058*	
C19F	0.5628 (5)	0.4752 (5)	0.6258 (3)	0.051 (3)	
H19F	0.5568	0.4486	0.6509	0.061*	
C19G	0.4900 (5)	0.0453 (4)	0.6247 (3)	0.046 (2)	
H19G	0.4703	0.0330	0.6501	0.055*	
C19H	0.6219 (5)	0.3492 (5)	0.3684 (3)	0.052 (3)	
H19H	0.6033	0.3228	0.3431	0.062*	
C20A	0.3750 (4)	0.5822 (4)	0.4148 (3)	0.040 (2)	
C20B	0.2110 (5)	0.2860 (4)	0.5789 (3)	0.043 (2)	
C20C	0.0434 (4)	0.4102 (4)	0.4157 (3)	0.042 (2)	
C20D	0.2902 (4)	0.7053 (4)	0.5797 (3)	0.041 (2)	
C20E	0.2946 (4)	0.1604 (4)	0.4154 (3)	0.041 (2)	
C20F	0.5406 (4)	0.4554 (4)	0.5797 (3)	0.041 (2)	
C20G	0.4582 (5)	0.0340 (4)	0.5783 (3)	0.043 (2)	
C20H	0.6224 (4)	0.3294 (4)	0.4144 (3)	0.042 (2)	
C21A	0.3988 (6)	0.6825 (6)	0.3086 (4)	0.069 (3)	
H21A	0.4299	0.6583	0.2946	0.103*	
H21B	0.4095	0.7274	0.3084	0.103*	
H21C	0.3556	0.6757	0.2905	0.103*	
C21B	0.3374 (6)	0.3366 (6)	0.6853 (3)	0.067 (3)	
H21D	0.3608	0.3766	0.6879	0.100*	
H21E	0.3676	0.3019	0.6947	0.100*	
H21F	0.3054	0.3374	0.7059	0.100*	
C21C	0.1183 (6)	0.4619 (5)	0.3094 (3)	0.066 (3)	
H21G	0.0794	0.4806	0.2910	0.099*	
H21H	0.1272	0.4216	0.2954	0.099*	
H21I	0.1546	0.4906	0.3095	0.099*	
C21D	0.3658 (5)	0.8062 (6)	0.6852 (3)	0.065 (3)	
H21J	0.3633	0.7715	0.7071	0.097*	
H21K	0.3382	0.8409	0.6917	0.097*	
H21L	0.4103	0.8211	0.6890	0.097*	
C21E	0.3668 (6)	0.2130 (6)	0.3094 (3)	0.067 (3)	
H21M	0.3714	0.1725	0.2941	0.100*	
H21N	0.4052	0.2387	0.3091	0.100*	
H21O	0.3290	0.2352	0.2925	0.100*	
C21F	0.6168 (6)	0.5564 (6)	0.6861 (3)	0.068 (3)	
H21P	0.6191	0.5206	0.7077	0.102*	
H21Q	0.5867	0.5878	0.6941	0.102*	
H21R	0.6595	0.5755	0.6888	0.102*	
C21G	0.5856 (6)	0.0848 (5)	0.6846 (4)	0.065 (3)	
H21S	0.6158	0.0501	0.6939	0.097*	
H21T	0.5541	0.0859	0.7055	0.097*	
H21U	0.6090	0.1248	0.6868	0.097*	
C21H	0.6459 (6)	0.4304 (5)	0.3084 (3)	0.061 (3)	
H21V	0.6428	0.4765	0.3077	0.092*	
H21W	0.6084	0.4123	0.2880	0.092*	
H21X	0.6850	0.4172	0.2976	0.092*	
O1A	0.3456 (3)	0.5227 (3)	0.4200 (2)	0.0495 (16)	
O1B	0.1516 (3)	0.2557 (3)	0.5742 (2)	0.0525 (17)	
O1C	−0.0141 (3)	0.3802 (3)	0.4205 (2)	0.0521 (17)	
O1D	0.2594 (3)	0.6466 (3)	0.5748 (2)	0.0492 (16)	
O1E	0.2364 (3)	0.1303 (3)	0.4205 (2)	0.0518 (17)	
O1F	0.5089 (3)	0.3967 (3)	0.5746 (2)	0.0519 (17)	
O1G	0.3984 (3)	0.0045 (3)	0.5736 (2)	0.0512 (17)	
O1H	0.5942 (3)	0.2701 (3)	0.4193 (2)	0.0510 (17)	
O2A	0.3168 (4)	0.4455 (3)	0.4645 (3)	0.066 (2)	
O2B	0.0634 (4)	0.2145 (4)	0.5297 (3)	0.070 (2)	
O2C	−0.0795 (3)	0.3402 (4)	0.4646 (3)	0.068 (2)	
O2D	0.2083 (4)	0.5682 (3)	0.5307 (3)	0.069 (2)	
O2E	0.1714 (4)	0.0896 (4)	0.4648 (3)	0.067 (2)	
O2F	0.4585 (4)	0.3192 (3)	0.5300 (3)	0.068 (2)	
O2G	0.3107 (3)	−0.0357 (4)	0.5294 (3)	0.067 (2)	
O2H	0.5656 (4)	0.1929 (3)	0.4639 (3)	0.069 (2)	
O3A	0.3892 (4)	0.5651 (3)	0.6752 (2)	0.0563 (18)	
O3B	0.1271 (4)	0.2372 (3)	0.3187 (2)	0.063 (2)	
O3C	0.0900 (4)	0.3617 (3)	0.6757 (2)	0.0607 (19)	
O3D	0.1741 (4)	0.6866 (3)	0.3197 (2)	0.0609 (19)	
O3E	0.3408 (4)	0.1111 (4)	0.6760 (2)	0.064 (2)	
O3F	0.4252 (3)	0.4416 (3)	0.3196 (2)	0.0564 (18)	
O3G	0.3753 (4)	−0.0138 (3)	0.3181 (2)	0.0615 (19)	
O3H	0.6380 (4)	0.3114 (4)	0.6749 (2)	0.0610 (19)	

### Atomic displacement parameters (Å^2^)

**Table:**

	*U*^11^	*U*^22^	*U*^33^	*U*^12^	*U*^13^	*U*^23^
C1A	0.031 (5)	0.048 (6)	0.058 (7)	−0.009 (4)	−0.005 (5)	−0.007 (5)
C1B	0.049 (6)	0.037 (5)	0.061 (7)	0.000 (4)	0.021 (5)	0.011 (5)
C1C	0.042 (6)	0.042 (5)	0.055 (7)	−0.010 (4)	0.009 (5)	−0.007 (4)
C1D	0.045 (6)	0.039 (5)	0.053 (7)	−0.005 (4)	0.019 (5)	0.012 (4)
C1E	0.039 (6)	0.036 (5)	0.057 (7)	−0.008 (4)	0.003 (5)	−0.013 (4)
C1F	0.040 (5)	0.045 (5)	0.063 (7)	−0.002 (4)	0.022 (5)	0.012 (5)
C1G	0.044 (6)	0.041 (5)	0.047 (6)	−0.003 (4)	0.013 (5)	0.005 (4)
C1H	0.036 (5)	0.036 (5)	0.055 (7)	−0.009 (4)	−0.001 (5)	−0.006 (4)
C2A	0.026 (4)	0.045 (5)	0.033 (5)	0.001 (4)	0.002 (4)	0.001 (4)
C2B	0.038 (5)	0.028 (4)	0.043 (5)	0.001 (4)	0.004 (4)	0.006 (4)
C2C	0.038 (5)	0.036 (4)	0.046 (6)	−0.003 (4)	0.012 (4)	−0.004 (4)
C2D	0.032 (5)	0.047 (5)	0.033 (5)	−0.002 (4)	0.012 (4)	0.002 (4)
C2E	0.040 (5)	0.035 (4)	0.037 (5)	0.003 (4)	0.010 (4)	−0.006 (4)
C2F	0.030 (5)	0.050 (5)	0.042 (6)	0.005 (4)	0.012 (4)	0.004 (4)
C2G	0.038 (5)	0.033 (4)	0.041 (5)	−0.001 (4)	0.003 (4)	0.007 (4)
C2H	0.030 (5)	0.042 (5)	0.035 (5)	−0.002 (4)	0.003 (4)	0.001 (4)
C3A	0.033 (5)	0.040 (5)	0.058 (7)	−0.009 (4)	0.012 (5)	0.012 (4)
C3B	0.034 (5)	0.036 (5)	0.059 (7)	−0.010 (4)	0.001 (5)	−0.002 (4)
C3C	0.047 (6)	0.037 (5)	0.055 (7)	−0.006 (4)	0.022 (5)	0.000 (4)
C3D	0.037 (5)	0.037 (5)	0.047 (6)	−0.009 (4)	0.010 (4)	−0.011 (4)
C3E	0.039 (5)	0.031 (5)	0.054 (7)	−0.004 (4)	0.016 (5)	0.003 (4)
C3F	0.029 (5)	0.042 (5)	0.055 (7)	−0.006 (4)	0.006 (4)	−0.012 (5)
C3G	0.038 (5)	0.033 (4)	0.050 (6)	−0.006 (4)	0.007 (4)	−0.005 (4)
C3H	0.032 (5)	0.046 (5)	0.046 (6)	−0.014 (4)	0.006 (4)	0.007 (4)
C4A	0.031 (5)	0.042 (5)	0.037 (5)	−0.005 (4)	0.009 (4)	0.008 (4)
C4B	0.041 (5)	0.031 (4)	0.040 (5)	0.001 (4)	−0.003 (4)	−0.003 (4)
C4C	0.047 (6)	0.033 (4)	0.043 (5)	0.000 (4)	0.018 (5)	0.001 (4)
C4D	0.031 (5)	0.045 (5)	0.034 (5)	−0.007 (4)	0.003 (4)	−0.007 (4)
C4E	0.043 (5)	0.038 (5)	0.035 (5)	0.004 (4)	0.012 (4)	0.002 (4)
C4F	0.031 (5)	0.046 (5)	0.041 (5)	−0.004 (4)	0.007 (4)	−0.007 (4)
C4G	0.047 (6)	0.034 (4)	0.040 (5)	0.000 (4)	−0.001 (4)	−0.002 (4)
C4H	0.034 (5)	0.049 (5)	0.037 (5)	0.001 (4)	0.011 (4)	0.005 (4)
C5A	0.049 (6)	0.055 (6)	0.039 (5)	−0.002 (5)	0.017 (4)	0.010 (4)
C5B	0.046 (6)	0.046 (5)	0.055 (6)	−0.008 (4)	0.001 (5)	−0.008 (4)
C5C	0.064 (7)	0.048 (6)	0.053 (6)	−0.010 (5)	0.021 (5)	0.014 (5)
C5D	0.053 (6)	0.059 (6)	0.039 (6)	−0.004 (5)	−0.001 (5)	−0.005 (4)
C5E	0.053 (6)	0.052 (6)	0.046 (6)	−0.001 (5)	0.011 (5)	0.008 (4)
C5F	0.045 (6)	0.056 (6)	0.042 (6)	−0.011 (5)	0.000 (4)	−0.002 (4)
C5G	0.047 (6)	0.055 (6)	0.046 (6)	−0.002 (5)	0.006 (5)	−0.012 (4)
C5H	0.065 (7)	0.052 (6)	0.037 (5)	−0.005 (5)	0.018 (5)	0.006 (4)
C6A	0.060 (6)	0.047 (5)	0.036 (5)	0.007 (5)	0.013 (5)	0.006 (4)
C6B	0.059 (6)	0.039 (5)	0.035 (5)	−0.001 (4)	−0.003 (4)	−0.001 (4)
C6C	0.058 (6)	0.039 (5)	0.038 (5)	0.000 (4)	0.017 (5)	0.005 (4)
C6D	0.047 (5)	0.046 (5)	0.030 (5)	0.003 (4)	0.008 (4)	0.002 (4)
C6E	0.044 (6)	0.058 (6)	0.041 (6)	0.011 (5)	0.011 (4)	−0.001 (4)
C6F	0.044 (5)	0.050 (5)	0.038 (5)	0.006 (4)	0.002 (4)	−0.005 (4)
C6G	0.049 (6)	0.055 (6)	0.038 (5)	−0.001 (5)	0.005 (4)	0.002 (4)
C6H	0.048 (6)	0.051 (5)	0.034 (5)	0.008 (5)	0.008 (4)	0.006 (4)
C7A	0.091 (9)	0.056 (6)	0.040 (6)	0.007 (6)	0.009 (6)	0.001 (5)
C7B	0.081 (8)	0.080 (8)	0.027 (5)	0.016 (7)	−0.002 (5)	−0.007 (5)
C7C	0.077 (8)	0.063 (7)	0.033 (6)	0.011 (6)	0.011 (5)	0.012 (5)
C7D	0.066 (7)	0.062 (7)	0.034 (6)	0.006 (5)	0.006 (5)	−0.002 (5)
C7E	0.065 (8)	0.091 (8)	0.029 (5)	0.009 (7)	0.004 (5)	0.002 (5)
C7F	0.078 (8)	0.064 (7)	0.035 (6)	0.001 (6)	0.001 (5)	−0.006 (5)
C7G	0.072 (8)	0.088 (8)	0.033 (6)	0.002 (7)	0.004 (5)	−0.005 (5)
C7H	0.063 (7)	0.074 (7)	0.033 (6)	0.003 (6)	0.008 (5)	0.001 (5)
C8A	0.096 (9)	0.050 (6)	0.035 (6)	0.010 (6)	−0.006 (6)	−0.001 (5)
C8B	0.057 (7)	0.086 (8)	0.035 (6)	0.010 (6)	0.012 (5)	0.008 (5)
C8C	0.070 (8)	0.072 (7)	0.035 (6)	−0.001 (6)	0.005 (5)	−0.007 (5)
C8D	0.076 (8)	0.069 (7)	0.038 (6)	0.004 (6)	0.014 (6)	0.011 (5)
C8E	0.047 (7)	0.116 (10)	0.034 (6)	0.007 (7)	−0.003 (5)	−0.014 (6)
C8F	0.102 (10)	0.053 (6)	0.035 (6)	0.009 (6)	0.019 (6)	0.004 (5)
C8G	0.054 (7)	0.096 (9)	0.044 (7)	0.007 (6)	0.014 (5)	0.016 (6)
C8H	0.076 (8)	0.070 (7)	0.034 (6)	−0.002 (6)	−0.014 (5)	−0.005 (5)
C9A	0.068 (7)	0.039 (5)	0.071 (8)	0.008 (5)	−0.017 (6)	0.004 (5)
C9B	0.045 (6)	0.064 (7)	0.055 (7)	−0.002 (5)	0.012 (5)	0.011 (5)
C9C	0.043 (6)	0.072 (7)	0.056 (7)	0.000 (5)	0.008 (5)	−0.011 (5)
C9D	0.083 (8)	0.050 (6)	0.051 (7)	0.000 (6)	0.031 (6)	0.005 (5)
C9E	0.031 (5)	0.097 (8)	0.050 (6)	−0.006 (5)	0.004 (5)	−0.021 (6)
C9F	0.082 (8)	0.046 (6)	0.061 (7)	0.000 (5)	0.031 (6)	−0.002 (5)
C9G	0.040 (6)	0.082 (8)	0.052 (7)	0.002 (5)	0.011 (5)	0.014 (6)
C9H	0.069 (7)	0.049 (6)	0.048 (6)	0.006 (5)	−0.012 (5)	−0.009 (5)
C10A	0.052 (6)	0.033 (5)	0.043 (6)	−0.001 (4)	−0.003 (4)	−0.001 (4)
C10B	0.045 (5)	0.048 (5)	0.038 (5)	0.006 (4)	0.001 (4)	0.005 (4)
C10C	0.058 (6)	0.043 (5)	0.036 (5)	0.001 (4)	0.016 (4)	−0.004 (4)
C10D	0.063 (6)	0.035 (5)	0.038 (5)	0.003 (4)	0.016 (5)	0.003 (4)
C10E	0.039 (5)	0.059 (6)	0.041 (5)	0.003 (4)	0.006 (4)	−0.013 (4)
C10F	0.064 (7)	0.038 (5)	0.044 (6)	−0.003 (5)	0.015 (5)	−0.003 (4)
C10G	0.040 (5)	0.054 (6)	0.039 (5)	0.004 (4)	0.004 (4)	0.010 (4)
C10H	0.051 (6)	0.047 (5)	0.039 (6)	0.004 (5)	−0.001 (4)	0.006 (4)
C11A	0.043 (5)	0.034 (4)	0.033 (5)	0.000 (4)	0.004 (4)	0.003 (3)
C11B	0.045 (5)	0.030 (4)	0.032 (5)	0.008 (4)	0.001 (4)	−0.001 (3)
C11C	0.047 (5)	0.035 (4)	0.036 (5)	0.009 (4)	0.017 (4)	0.002 (4)
C11D	0.036 (5)	0.035 (4)	0.029 (4)	0.004 (4)	0.008 (4)	0.005 (3)
C11E	0.041 (5)	0.047 (5)	0.038 (5)	0.012 (4)	0.009 (4)	−0.005 (4)
C11F	0.030 (4)	0.036 (4)	0.033 (5)	0.000 (4)	0.000 (4)	−0.003 (3)
C11G	0.035 (5)	0.042 (5)	0.039 (5)	0.012 (4)	−0.001 (4)	0.008 (4)
C11H	0.037 (5)	0.038 (4)	0.033 (5)	0.008 (4)	0.004 (4)	0.004 (4)
C12A	0.026 (4)	0.041 (4)	0.033 (5)	0.004 (4)	0.003 (4)	0.003 (4)
C12B	0.038 (5)	0.027 (4)	0.036 (5)	−0.001 (4)	−0.006 (4)	−0.004 (3)
C12C	0.043 (5)	0.026 (4)	0.035 (5)	0.001 (4)	0.014 (4)	0.002 (3)
C12D	0.036 (5)	0.041 (5)	0.028 (4)	0.004 (4)	0.011 (4)	0.005 (4)
C12E	0.038 (5)	0.033 (4)	0.039 (5)	0.004 (4)	0.012 (4)	−0.002 (4)
C12F	0.030 (4)	0.038 (4)	0.033 (5)	0.000 (4)	0.007 (4)	−0.003 (4)
C12G	0.035 (5)	0.036 (4)	0.036 (5)	0.003 (4)	0.000 (4)	0.003 (4)
C12H	0.028 (4)	0.040 (5)	0.034 (5)	−0.002 (4)	0.006 (4)	−0.001 (4)
C13A	0.026 (4)	0.032 (4)	0.035 (5)	−0.002 (3)	0.003 (3)	−0.001 (3)
C13B	0.034 (5)	0.032 (4)	0.035 (5)	0.000 (4)	−0.001 (4)	0.004 (3)
C13C	0.041 (5)	0.029 (4)	0.035 (5)	0.000 (4)	0.017 (4)	−0.001 (3)
C13D	0.036 (5)	0.034 (4)	0.031 (5)	0.001 (4)	0.012 (4)	0.005 (3)
C13E	0.036 (5)	0.035 (4)	0.034 (5)	−0.002 (4)	0.011 (4)	−0.005 (3)
C13F	0.040 (5)	0.033 (4)	0.035 (5)	−0.005 (4)	0.009 (4)	0.001 (3)
C13G	0.033 (4)	0.035 (4)	0.037 (5)	0.005 (4)	0.002 (4)	0.004 (4)
C13H	0.031 (4)	0.037 (4)	0.032 (5)	−0.001 (4)	−0.001 (4)	−0.002 (3)
C14A	0.020 (4)	0.035 (4)	0.038 (5)	0.002 (3)	0.003 (3)	−0.002 (3)
C14B	0.041 (5)	0.025 (4)	0.046 (5)	0.001 (4)	0.005 (4)	0.004 (4)
C14C	0.049 (5)	0.028 (4)	0.030 (5)	0.002 (4)	0.012 (4)	−0.004 (3)
C14D	0.026 (4)	0.035 (4)	0.034 (5)	0.002 (3)	0.013 (4)	0.004 (3)
C14E	0.042 (5)	0.032 (4)	0.038 (5)	0.001 (4)	0.013 (4)	−0.004 (4)
C14F	0.029 (4)	0.038 (5)	0.043 (5)	0.005 (4)	0.013 (4)	0.004 (4)
C14G	0.035 (5)	0.034 (4)	0.032 (5)	0.006 (4)	0.000 (4)	0.009 (3)
C14H	0.026 (4)	0.042 (5)	0.032 (5)	0.003 (4)	0.000 (3)	−0.001 (3)
C15A	0.028 (4)	0.044 (5)	0.031 (5)	0.003 (4)	0.001 (4)	0.001 (4)
C15B	0.048 (5)	0.024 (4)	0.038 (5)	0.007 (4)	0.002 (4)	0.000 (3)
C15C	0.047 (5)	0.027 (4)	0.034 (5)	0.005 (4)	0.011 (4)	0.001 (3)
C15D	0.032 (5)	0.045 (5)	0.032 (5)	0.004 (4)	0.013 (4)	0.000 (4)
C15E	0.043 (5)	0.024 (4)	0.033 (5)	0.003 (4)	0.007 (4)	−0.002 (3)
C15F	0.038 (5)	0.050 (5)	0.032 (5)	0.010 (4)	0.013 (4)	−0.001 (4)
C15G	0.044 (5)	0.025 (4)	0.035 (5)	0.007 (4)	0.004 (4)	0.001 (3)
C15H	0.030 (5)	0.045 (5)	0.029 (5)	0.002 (4)	0.000 (4)	0.000 (4)
C16A	0.042 (5)	0.050 (5)	0.029 (5)	−0.004 (4)	0.001 (4)	0.005 (4)
C16B	0.049 (6)	0.039 (5)	0.031 (5)	−0.008 (4)	0.001 (4)	0.007 (4)
C16C	0.058 (6)	0.040 (5)	0.031 (5)	−0.006 (4)	0.011 (4)	−0.006 (4)
C16D	0.044 (6)	0.053 (6)	0.037 (5)	−0.005 (4)	0.014 (4)	−0.004 (4)
C16E	0.048 (6)	0.050 (5)	0.030 (5)	−0.001 (4)	0.008 (4)	−0.005 (4)
C16F	0.044 (6)	0.055 (6)	0.034 (5)	−0.005 (5)	0.011 (4)	−0.006 (4)
C16G	0.063 (7)	0.036 (5)	0.035 (5)	0.001 (4)	0.010 (5)	0.007 (4)
C16H	0.040 (5)	0.061 (6)	0.030 (5)	−0.002 (5)	0.000 (4)	0.006 (4)
C17A	0.045 (6)	0.050 (6)	0.044 (6)	−0.001 (5)	0.004 (5)	0.016 (4)
C17B	0.052 (6)	0.052 (6)	0.041 (6)	−0.004 (5)	−0.008 (5)	0.002 (4)
C17C	0.055 (6)	0.042 (5)	0.040 (6)	0.000 (4)	0.017 (5)	0.005 (4)
C17D	0.044 (6)	0.057 (6)	0.040 (6)	−0.005 (5)	0.006 (4)	−0.013 (5)
C17E	0.055 (6)	0.055 (6)	0.040 (6)	0.001 (5)	0.019 (5)	0.003 (5)
C17F	0.049 (6)	0.061 (6)	0.034 (5)	−0.006 (5)	0.008 (5)	−0.014 (5)
C17G	0.057 (6)	0.041 (5)	0.041 (6)	−0.007 (5)	−0.003 (5)	−0.006 (4)
C17H	0.041 (5)	0.066 (6)	0.038 (6)	−0.002 (5)	0.009 (5)	0.023 (5)
C18A	0.051 (6)	0.066 (6)	0.031 (5)	0.020 (5)	0.009 (4)	0.006 (5)
C18B	0.065 (7)	0.048 (5)	0.038 (6)	0.015 (5)	0.002 (5)	−0.004 (4)
C18C	0.070 (7)	0.041 (5)	0.035 (5)	0.017 (5)	0.020 (5)	0.006 (4)
C18D	0.048 (6)	0.063 (6)	0.032 (5)	0.019 (5)	0.009 (4)	−0.006 (4)
C18E	0.061 (6)	0.054 (6)	0.028 (5)	0.015 (5)	0.008 (5)	−0.006 (4)
C18F	0.046 (6)	0.067 (7)	0.038 (6)	0.020 (5)	0.012 (5)	−0.006 (5)
C18G	0.070 (7)	0.039 (5)	0.037 (5)	0.012 (5)	0.001 (5)	0.000 (4)
C18H	0.038 (5)	0.069 (6)	0.028 (5)	0.012 (5)	0.004 (4)	0.008 (4)
C19A	0.044 (6)	0.069 (7)	0.035 (5)	0.022 (5)	−0.004 (4)	−0.011 (5)
C19B	0.069 (7)	0.055 (6)	0.033 (5)	0.023 (5)	0.011 (5)	0.008 (4)
C19C	0.064 (7)	0.047 (5)	0.033 (5)	0.016 (5)	0.003 (5)	0.001 (4)
C19D	0.052 (6)	0.066 (6)	0.034 (5)	0.021 (5)	0.013 (4)	0.006 (5)
C19E	0.059 (6)	0.047 (5)	0.034 (5)	0.014 (5)	−0.006 (4)	−0.005 (4)
C19F	0.049 (6)	0.069 (7)	0.037 (5)	0.022 (5)	0.017 (5)	0.012 (5)
C19G	0.060 (6)	0.048 (5)	0.030 (5)	0.008 (5)	0.010 (4)	−0.001 (4)
C19H	0.049 (6)	0.079 (7)	0.025 (5)	0.015 (5)	0.002 (4)	−0.008 (5)
C20A	0.035 (5)	0.044 (5)	0.039 (5)	0.003 (4)	0.002 (4)	−0.004 (4)
C20B	0.049 (6)	0.036 (5)	0.046 (6)	0.005 (4)	0.013 (4)	0.006 (4)
C20C	0.044 (5)	0.038 (5)	0.044 (5)	0.003 (4)	0.010 (4)	−0.006 (4)
C20D	0.039 (5)	0.047 (5)	0.041 (5)	0.003 (4)	0.015 (4)	0.007 (4)
C20E	0.041 (5)	0.040 (5)	0.040 (5)	0.004 (4)	0.001 (4)	−0.009 (4)
C20F	0.037 (5)	0.050 (5)	0.040 (5)	0.009 (4)	0.014 (4)	0.005 (4)
C20G	0.049 (6)	0.039 (5)	0.041 (5)	0.007 (4)	0.008 (4)	0.007 (4)
C20H	0.036 (5)	0.049 (5)	0.040 (5)	0.007 (4)	0.003 (4)	−0.004 (4)
C21A	0.079 (8)	0.093 (9)	0.034 (6)	0.027 (7)	0.008 (5)	0.012 (5)
C21B	0.079 (8)	0.083 (8)	0.031 (6)	0.011 (7)	−0.007 (5)	−0.001 (5)
C21C	0.096 (9)	0.070 (7)	0.035 (6)	0.015 (7)	0.021 (6)	0.007 (5)
C21D	0.061 (7)	0.093 (9)	0.036 (6)	0.006 (6)	0.000 (5)	−0.016 (5)
C21E	0.079 (8)	0.091 (8)	0.032 (6)	0.011 (7)	0.013 (5)	0.003 (5)
C21F	0.087 (9)	0.084 (8)	0.028 (6)	0.013 (7)	−0.002 (5)	−0.013 (5)
C21G	0.084 (8)	0.066 (7)	0.039 (6)	0.012 (6)	−0.003 (6)	−0.006 (5)
C21H	0.067 (7)	0.088 (8)	0.031 (6)	0.014 (6)	0.014 (5)	0.016 (5)
O1A	0.052 (4)	0.058 (4)	0.035 (4)	−0.006 (3)	−0.001 (3)	−0.010 (3)
O1B	0.058 (5)	0.060 (4)	0.042 (4)	0.001 (4)	0.015 (3)	0.015 (3)
O1C	0.050 (4)	0.060 (4)	0.044 (4)	−0.012 (3)	0.001 (3)	−0.013 (3)
O1D	0.061 (4)	0.052 (4)	0.039 (4)	0.003 (3)	0.019 (3)	0.008 (3)
O1E	0.046 (4)	0.064 (4)	0.042 (4)	−0.005 (3)	−0.002 (3)	−0.018 (3)
O1F	0.062 (5)	0.054 (4)	0.043 (4)	0.004 (4)	0.019 (4)	0.009 (3)
O1G	0.050 (4)	0.064 (4)	0.042 (4)	−0.009 (3)	0.016 (3)	0.011 (3)
O1H	0.058 (4)	0.053 (4)	0.037 (4)	−0.008 (3)	−0.003 (3)	−0.006 (3)
O2A	0.072 (5)	0.062 (5)	0.057 (5)	−0.023 (4)	−0.004 (4)	−0.005 (4)
O2B	0.067 (5)	0.081 (5)	0.063 (5)	−0.021 (4)	0.017 (4)	0.016 (4)
O2C	0.057 (5)	0.075 (5)	0.069 (5)	−0.019 (4)	0.006 (4)	−0.018 (4)
O2D	0.092 (6)	0.060 (5)	0.061 (5)	−0.020 (4)	0.033 (4)	0.007 (4)
O2E	0.061 (5)	0.081 (5)	0.058 (5)	−0.026 (4)	0.006 (4)	−0.021 (4)
O2F	0.084 (6)	0.057 (4)	0.066 (5)	−0.014 (4)	0.025 (4)	0.002 (4)
O2G	0.060 (5)	0.079 (5)	0.065 (5)	−0.018 (4)	0.015 (4)	0.020 (4)
O2H	0.072 (5)	0.065 (5)	0.063 (5)	−0.022 (4)	−0.003 (4)	−0.008 (4)
O3A	0.068 (5)	0.059 (4)	0.045 (4)	−0.002 (4)	0.019 (4)	0.004 (3)
O3B	0.082 (5)	0.061 (4)	0.042 (4)	−0.019 (4)	0.002 (4)	−0.012 (3)
O3C	0.082 (5)	0.057 (4)	0.043 (4)	−0.007 (4)	0.013 (4)	0.014 (3)
O3D	0.065 (5)	0.075 (5)	0.039 (4)	−0.011 (4)	0.002 (3)	−0.002 (3)
O3E	0.077 (5)	0.076 (5)	0.039 (4)	−0.009 (4)	0.012 (4)	0.010 (3)
O3F	0.058 (4)	0.063 (4)	0.044 (4)	−0.011 (4)	−0.004 (3)	−0.005 (3)
O3G	0.069 (5)	0.068 (5)	0.047 (4)	−0.017 (4)	0.008 (4)	−0.017 (3)
O3H	0.071 (5)	0.078 (5)	0.035 (4)	−0.006 (4)	0.014 (3)	0.005 (3)

### Geometric parameters (Å, º)

**Table:**

C1A—O2A	1.205 (11)	C10A—C11A	1.370 (12)
C1A—O1A	1.358 (12)	C10A—H10A	0.9300
C1A—C2A	1.477 (12)	C10B—C11B	1.392 (12)
C1B—O2B	1.211 (11)	C10B—H10B	0.9300
C1B—O1B	1.367 (12)	C10C—C11C	1.379 (12)
C1B—C2B	1.460 (13)	C10C—H10C	0.9300
C1C—O2C	1.199 (11)	C10D—C11D	1.397 (12)
C1C—O1C	1.357 (12)	C10D—H10D	0.9300
C1C—C2C	1.458 (13)	C10E—C11E	1.385 (12)
C1D—O2D	1.223 (11)	C10E—H10E	0.9300
C1D—O1D	1.351 (12)	C10F—C11F	1.387 (12)
C1D—C2D	1.461 (12)	C10F—H10F	0.9300
C1E—O2E	1.211 (11)	C10G—C11G	1.403 (12)
C1E—O1E	1.352 (12)	C10G—H10G	0.9300
C1E—C2E	1.455 (13)	C10H—C11H	1.381 (12)
C1F—O2F	1.196 (11)	C10H—H10H	0.9300
C1F—O1F	1.364 (12)	C11A—C12A	1.466 (12)
C1F—C2F	1.477 (12)	C11B—C12B	1.475 (12)
C1G—O2G	1.212 (11)	C11C—C12C	1.466 (12)
C1G—O1G	1.359 (11)	C11D—C12D	1.472 (11)
C1G—C2G	1.446 (12)	C11E—C12E	1.473 (12)
C1H—O2H	1.205 (10)	C11F—C12F	1.475 (11)
C1H—O1H	1.358 (11)	C11G—C12G	1.466 (12)
C1H—C2H	1.458 (12)	C11H—C12H	1.459 (12)
C2A—C14A	1.384 (11)	C12A—C13A	1.399 (11)
C2A—C3A	1.403 (12)	C12B—C13B	1.399 (11)
C2B—C14B	1.375 (12)	C12C—C13C	1.406 (11)
C2B—C3B	1.407 (13)	C12D—C13D	1.387 (11)
C2C—C3C	1.397 (13)	C12E—C13E	1.392 (11)
C2C—C14C	1.397 (12)	C12F—C13F	1.387 (11)
C2D—C14D	1.386 (11)	C12G—C13G	1.394 (12)
C2D—C3D	1.396 (12)	C12H—C13H	1.402 (11)
C2E—C14E	1.391 (12)	C13A—C14A	1.405 (11)
C2E—C3E	1.409 (12)	C13A—H13A	0.9300
C2F—C14F	1.387 (12)	C13B—C14B	1.410 (12)
C2F—C3F	1.396 (13)	C13B—H13B	0.9300
C2G—C14G	1.388 (12)	C13C—C14C	1.398 (12)
C2G—C3G	1.407 (12)	C13C—H13C	0.9300
C2H—C14H	1.393 (11)	C13D—C14D	1.421 (10)
C2H—C3H	1.409 (12)	C13D—H13D	0.9300
C3A—C4A	1.357 (13)	C13E—C14E	1.401 (12)
C3A—H3A	0.9300	C13E—H13E	0.9300
C3B—C4B	1.382 (13)	C13F—C14F	1.420 (11)
C3B—H3B	0.9300	C13F—H13F	0.9300
C3C—C4C	1.363 (13)	C13G—C14G	1.407 (11)
C3C—H3C	0.9300	C13G—H13G	0.9300
C3D—C4D	1.387 (12)	C13H—C14H	1.412 (11)
C3D—H3D	0.9300	C13H—H13H	0.9300
C3E—C4E	1.378 (13)	C14A—C15A	1.451 (11)
C3E—H3E	0.9300	C14B—C15B	1.455 (12)
C3F—C4F	1.365 (13)	C14C—C15C	1.454 (11)
C3F—H3F	0.9300	C14D—C15D	1.455 (11)
C3G—C4G	1.376 (13)	C14E—C15E	1.458 (11)
C3G—H3G	0.9300	C14F—C15F	1.447 (12)
C3H—C4H	1.356 (13)	C14G—C15G	1.456 (12)
C3H—H3H	0.9300	C14H—C15H	1.449 (11)
C4A—C12A	1.399 (11)	C15A—C16A	1.394 (12)
C4A—C5A	1.500 (11)	C15A—C20A	1.402 (12)
C4B—C12B	1.384 (12)	C15B—C20B	1.379 (12)
C4B—C5B	1.504 (12)	C15B—C16B	1.398 (12)
C4C—C12C	1.389 (11)	C15C—C20C	1.398 (12)
C4C—C5C	1.485 (12)	C15C—C16C	1.402 (12)
C4D—C12D	1.388 (11)	C15D—C16D	1.382 (12)
C4D—C5D	1.490 (12)	C15D—C20D	1.399 (11)
C4E—C12E	1.399 (12)	C15E—C20E	1.382 (12)
C4E—C5E	1.489 (12)	C15E—C16E	1.385 (12)
C4F—C12F	1.398 (11)	C15F—C20F	1.393 (12)
C4F—C5F	1.505 (12)	C15F—C16F	1.409 (12)
C4G—C12G	1.390 (12)	C15G—C20G	1.395 (12)
C4G—C5G	1.480 (12)	C15G—C16G	1.395 (12)
C4H—C12H	1.396 (12)	C15H—C16H	1.378 (12)
C4H—C5H	1.501 (12)	C15H—C20H	1.403 (12)
C5A—O3A	1.446 (11)	C16A—C17A	1.385 (12)
C5A—H5A	0.9700	C16A—H16A	0.9300
C5A—H5B	0.9700	C16B—C17B	1.379 (12)
C5B—O3B	1.436 (12)	C16B—H16B	0.9300
C5B—H5C	0.9700	C16C—C17C	1.372 (12)
C5B—H5D	0.9700	C16C—H16C	0.9300
C5C—O3C	1.411 (12)	C16D—C17D	1.384 (12)
C5C—H5E	0.9700	C16D—H16D	0.9300
C5C—H5F	0.9700	C16E—C17E	1.386 (12)
C5D—O3D	1.433 (12)	C16E—H16E	0.9300
C5D—H5G	0.9700	C16F—C17F	1.384 (12)
C5D—H5H	0.9700	C16F—H16F	0.9300
C5E—O3E	1.427 (12)	C16G—C17G	1.380 (12)
C5E—H5I	0.9700	C16G—H16G	0.9300
C5E—H5J	0.9700	C16H—C17H	1.403 (12)
C5F—O3F	1.437 (11)	C16H—H16H	0.9300
C5F—H5K	0.9700	C17A—C18A	1.370 (14)
C5F—H5L	0.9700	C17A—H17A	0.9300
C5G—O3G	1.438 (11)	C17B—C18B	1.383 (14)
C5G—H5M	0.9700	C17B—H17B	0.9300
C5G—H5N	0.9700	C17C—C18C	1.398 (14)
C5H—O3H	1.448 (11)	C17C—H17C	0.9300
C5H—H5O	0.9700	C17D—C18D	1.405 (14)
C5H—H5P	0.9700	C17D—H17D	0.9300
C6A—O3A	1.381 (11)	C17E—C18E	1.357 (13)
C6A—C7A	1.385 (14)	C17E—H17E	0.9300
C6A—C11A	1.412 (12)	C17F—C18F	1.408 (14)
C6B—O3B	1.388 (11)	C17F—H17F	0.9300
C6B—C7B	1.396 (14)	C17G—C18G	1.397 (14)
C6B—C11B	1.402 (12)	C17G—H17G	0.9300
C6C—O3C	1.386 (11)	C17H—C18H	1.372 (14)
C6C—C11C	1.390 (11)	C17H—H17H	0.9300
C6C—C7C	1.394 (14)	C18A—C19A	1.391 (14)
C6D—O3D	1.355 (11)	C18A—C21A	1.508 (13)
C6D—C7D	1.384 (13)	C18B—C19B	1.396 (14)
C6D—C11D	1.388 (12)	C18B—C21B	1.490 (13)
C6E—O3E	1.361 (12)	C18C—C19C	1.399 (14)
C6E—C7E	1.408 (14)	C18C—C21C	1.511 (12)
C6E—C11E	1.409 (13)	C18D—C19D	1.371 (14)
C6F—O3F	1.374 (11)	C18D—C21D	1.497 (13)
C6F—C7F	1.386 (14)	C18E—C19E	1.383 (14)
C6F—C11F	1.396 (12)	C18E—C21E	1.504 (13)
C6G—O3G	1.351 (11)	C18F—C19F	1.379 (14)
C6G—C11G	1.391 (13)	C18F—C21F	1.498 (13)
C6G—C7G	1.403 (14)	C18G—C19G	1.378 (13)
C6H—O3H	1.362 (11)	C18G—C21G	1.514 (13)
C6H—C7H	1.383 (13)	C18H—C19H	1.367 (14)
C6H—C11H	1.400 (12)	C18H—C21H	1.509 (12)
C7A—C8A	1.345 (15)	C19A—C20A	1.369 (13)
C7A—H7A	0.9300	C19A—H19A	0.9300
C7B—C8B	1.351 (15)	C19B—C20B	1.395 (13)
C7B—H7B	0.9300	C19B—H19B	0.9300
C7C—C8C	1.368 (15)	C19C—C20C	1.383 (13)
C7C—H7C	0.9300	C19C—H19C	0.9300
C7D—C8D	1.366 (14)	C19D—C20D	1.371 (13)
C7D—H7D	0.9300	C19D—H19D	0.9300
C7E—C8E	1.349 (15)	C19E—C20E	1.383 (13)
C7E—H7E	0.9300	C19E—H19E	0.9300
C7F—C8F	1.346 (15)	C19F—C20F	1.370 (13)
C7F—H7F	0.9300	C19F—H19F	0.9300
C7G—C8G	1.366 (15)	C19G—C20G	1.383 (12)
C7G—H7G	0.9300	C19G—H19G	0.9300
C7H—C8H	1.343 (15)	C19H—C20H	1.369 (12)
C7H—H7H	0.9300	C19H—H19H	0.9300
C8A—C9A	1.369 (16)	C20A—O1A	1.398 (11)
C8A—H8A	0.9300	C20B—O1B	1.368 (11)
C8B—C9B	1.368 (15)	C20C—O1C	1.375 (11)
C8B—H8B	0.9300	C20D—O1D	1.372 (11)
C8C—C9C	1.379 (14)	C20E—O1E	1.391 (11)
C8C—H8C	0.9300	C20F—O1F	1.380 (11)
C8D—C9D	1.377 (15)	C20G—O1G	1.369 (11)
C8D—H8D	0.9300	C20H—O1H	1.382 (11)
C8E—C9E	1.388 (15)	C21A—H21A	0.9600
C8E—H8E	0.9300	C21A—H21B	0.9600
C8F—C9F	1.370 (16)	C21A—H21C	0.9600
C8F—H8F	0.9300	C21B—H21D	0.9600
C8G—C9G	1.371 (15)	C21B—H21E	0.9600
C8G—H8G	0.9300	C21B—H21F	0.9600
C8H—C9H	1.391 (15)	C21C—H21G	0.9600
C8H—H8H	0.9300	C21C—H21H	0.9600
C9A—C10A	1.407 (14)	C21C—H21I	0.9600
C9A—H9A	0.9300	C21D—H21J	0.9600
C9B—C10B	1.384 (13)	C21D—H21K	0.9600
C9B—H9B	0.9300	C21D—H21L	0.9600
C9C—C10C	1.394 (13)	C21E—H21M	0.9600
C9C—H9C	0.9300	C21E—H21N	0.9600
C9D—C10D	1.418 (12)	C21E—H21O	0.9600
C9D—H9D	0.9300	C21F—H21P	0.9600
C9E—C10E	1.391 (13)	C21F—H21Q	0.9600
C9E—H9E	0.9300	C21F—H21R	0.9600
C9F—C10F	1.412 (14)	C21G—H21S	0.9600
C9F—H9F	0.9300	C21G—H21T	0.9600
C9G—C10G	1.394 (13)	C21G—H21U	0.9600
C9G—H9G	0.9300	C21H—H21V	0.9600
C9H—C10H	1.396 (13)	C21H—H21W	0.9600
C9H—H9H	0.9300	C21H—H21X	0.9600
			
O2A—C1A—O1A	117.5 (9)	C10G—C11G—C12G	123.8 (8)
O2A—C1A—C2A	124.9 (10)	C10H—C11H—C6H	117.0 (8)
O1A—C1A—C2A	117.6 (8)	C10H—C11H—C12H	124.1 (8)
O2B—C1B—O1B	117.0 (9)	C6H—C11H—C12H	118.9 (8)
O2B—C1B—C2B	125.4 (10)	C4A—C12A—C13A	119.0 (8)
O1B—C1B—C2B	117.6 (8)	C4A—C12A—C11A	118.2 (7)
O2C—C1C—O1C	116.5 (9)	C13A—C12A—C11A	122.8 (8)
O2C—C1C—C2C	125.1 (10)	C4B—C12B—C13B	118.9 (8)
O1C—C1C—C2C	118.5 (8)	C4B—C12B—C11B	118.5 (8)
O2D—C1D—O1D	117.8 (8)	C13B—C12B—C11B	122.6 (8)
O2D—C1D—C2D	123.7 (9)	C4C—C12C—C13C	118.9 (8)
O1D—C1D—C2D	118.4 (8)	C4C—C12C—C11C	118.4 (7)
O2E—C1E—O1E	116.4 (9)	C13C—C12C—C11C	122.7 (8)
O2E—C1E—C2E	124.6 (10)	C13D—C12D—C4D	119.7 (7)
O1E—C1E—C2E	118.9 (8)	C13D—C12D—C11D	122.9 (7)
O2F—C1F—O1F	117.7 (9)	C4D—C12D—C11D	117.4 (7)
O2F—C1F—C2F	125.2 (10)	C13E—C12E—C4E	119.4 (8)
O1F—C1F—C2F	117.1 (8)	C13E—C12E—C11E	123.4 (8)
O2G—C1G—O1G	116.0 (9)	C4E—C12E—C11E	117.2 (8)
O2G—C1G—C2G	124.8 (9)	C13F—C12F—C4F	119.7 (8)
O1G—C1G—C2G	119.2 (8)	C13F—C12F—C11F	122.6 (7)
O2H—C1H—O1H	117.5 (9)	C4F—C12F—C11F	117.7 (7)
O2H—C1H—C2H	124.2 (9)	C13G—C12G—C4G	119.5 (8)
O1H—C1H—C2H	118.2 (8)	C13G—C12G—C11G	123.3 (8)
C14A—C2A—C3A	120.1 (8)	C4G—C12G—C11G	117.1 (8)
C14A—C2A—C1A	121.6 (8)	C4H—C12H—C13H	118.9 (8)
C3A—C2A—C1A	118.3 (8)	C4H—C12H—C11H	118.1 (8)
C14B—C2B—C3B	121.6 (8)	C13H—C12H—C11H	123.0 (7)
C14B—C2B—C1B	121.5 (9)	C12A—C13A—C14A	120.1 (8)
C3B—C2B—C1B	116.9 (8)	C12A—C13A—H13A	119.9
C3C—C2C—C14C	119.5 (9)	C14A—C13A—H13A	119.9
C3C—C2C—C1C	119.2 (9)	C12B—C13B—C14B	120.6 (8)
C14C—C2C—C1C	121.3 (8)	C12B—C13B—H13B	119.7
C14D—C2D—C3D	121.1 (8)	C14B—C13B—H13B	119.7
C14D—C2D—C1D	120.7 (8)	C14C—C13C—C12C	120.7 (8)
C3D—C2D—C1D	118.1 (8)	C14C—C13C—H13C	119.6
C14E—C2E—C3E	120.3 (8)	C12C—C13C—H13C	119.6
C14E—C2E—C1E	121.2 (8)	C12D—C13D—C14D	120.0 (8)
C3E—C2E—C1E	118.5 (9)	C12D—C13D—H13D	120.0
C14F—C2F—C3F	120.4 (8)	C14D—C13D—H13D	120.0
C14F—C2F—C1F	121.3 (9)	C12E—C13E—C14E	121.1 (8)
C3F—C2F—C1F	118.3 (9)	C12E—C13E—H13E	119.4
C14G—C2G—C3G	119.7 (8)	C14E—C13E—H13E	119.4
C14G—C2G—C1G	121.3 (8)	C12F—C13F—C14F	120.0 (8)
C3G—C2G—C1G	119.0 (8)	C12F—C13F—H13F	120.0
C14H—C2H—C3H	119.9 (8)	C14F—C13F—H13F	120.0
C14H—C2H—C1H	121.3 (8)	C12G—C13G—C14G	120.2 (8)
C3H—C2H—C1H	118.9 (8)	C12G—C13G—H13G	119.9
C4A—C3A—C2A	120.3 (8)	C14G—C13G—H13G	119.9
C4A—C3A—H3A	119.8	C12H—C13H—C14H	120.2 (8)
C2A—C3A—H3A	119.8	C12H—C13H—H13H	119.9
C4B—C3B—C2B	118.5 (8)	C14H—C13H—H13H	119.9
C4B—C3B—H3B	120.7	C2A—C14A—C13A	119.3 (8)
C2B—C3B—H3B	120.7	C2A—C14A—C15A	118.4 (7)
C4C—C3C—C2C	121.2 (9)	C13A—C14A—C15A	122.2 (7)
C4C—C3C—H3C	119.4	C2B—C14B—C13B	118.6 (8)
C2C—C3C—H3C	119.4	C2B—C14B—C15B	118.0 (8)
C4D—C3D—C2D	118.9 (8)	C13B—C14B—C15B	123.4 (8)
C4D—C3D—H3D	120.5	C2C—C14C—C13C	119.0 (8)
C2D—C3D—H3D	120.5	C2C—C14C—C15C	117.9 (8)
C4E—C3E—C2E	120.2 (8)	C13C—C14C—C15C	123.0 (8)
C4E—C3E—H3E	119.9	C2D—C14D—C13D	118.9 (8)
C2E—C3E—H3E	119.9	C2D—C14D—C15D	118.4 (7)
C4F—C3F—C2F	120.4 (8)	C13D—C14D—C15D	122.7 (7)
C4F—C3F—H3F	119.8	C2E—C14E—C13E	118.8 (8)
C2F—C3F—H3F	119.8	C2E—C14E—C15E	117.4 (8)
C4G—C3G—C2G	120.1 (8)	C13E—C14E—C15E	123.8 (8)
C4G—C3G—H3G	119.9	C2F—C14F—C13F	118.8 (8)
C2G—C3G—H3G	119.9	C2F—C14F—C15F	118.5 (8)
C4H—C3H—C2H	120.2 (8)	C13F—C14F—C15F	122.7 (8)
C4H—C3H—H3H	119.9	C2G—C14G—C13G	119.6 (8)
C2H—C3H—H3H	119.9	C2G—C14G—C15G	117.4 (8)
C3A—C4A—C12A	121.0 (8)	C13G—C14G—C15G	122.9 (8)
C3A—C4A—C5A	121.2 (8)	C2H—C14H—C13H	119.2 (8)
C12A—C4A—C5A	117.8 (8)	C2H—C14H—C15H	118.2 (8)
C12B—C4B—C3B	121.7 (8)	C13H—C14H—C15H	122.5 (8)
C12B—C4B—C5B	118.1 (8)	C16A—C15A—C20A	115.3 (8)
C3B—C4B—C5B	120.1 (8)	C16A—C15A—C14A	125.7 (8)
C3C—C4C—C12C	120.6 (8)	C20A—C15A—C14A	119.0 (8)
C3C—C4C—C5C	122.4 (9)	C20B—C15B—C16B	116.6 (8)
C12C—C4C—C5C	117.0 (9)	C20B—C15B—C14B	118.9 (8)
C3D—C4D—C12D	121.3 (8)	C16B—C15B—C14B	124.5 (8)
C3D—C4D—C5D	121.8 (8)	C20C—C15C—C16C	116.8 (8)
C12D—C4D—C5D	116.9 (8)	C20C—C15C—C14C	118.3 (8)
C3E—C4E—C12E	120.2 (8)	C16C—C15C—C14C	124.9 (8)
C3E—C4E—C5E	121.0 (8)	C16D—C15D—C20D	116.0 (8)
C12E—C4E—C5E	118.7 (8)	C16D—C15D—C14D	125.4 (8)
C3F—C4F—C12F	120.6 (8)	C20D—C15D—C14D	118.6 (8)
C3F—C4F—C5F	121.9 (8)	C20E—C15E—C16E	116.3 (8)
C12F—C4F—C5F	117.5 (8)	C20E—C15E—C14E	119.5 (8)
C3G—C4G—C12G	120.8 (8)	C16E—C15E—C14E	124.1 (8)
C3G—C4G—C5G	121.2 (9)	C20F—C15F—C16F	116.0 (8)
C12G—C4G—C5G	118.0 (8)	C20F—C15F—C14F	119.0 (8)
C3H—C4H—C12H	121.6 (8)	C16F—C15F—C14F	125.0 (8)
C3H—C4H—C5H	121.4 (8)	C20G—C15G—C16G	116.1 (8)
C12H—C4H—C5H	117.0 (8)	C20G—C15G—C14G	119.0 (8)
O3A—C5A—C4A	112.2 (7)	C16G—C15G—C14G	124.9 (8)
O3A—C5A—H5A	109.2	C16H—C15H—C20H	115.8 (8)
C4A—C5A—H5A	109.2	C16H—C15H—C14H	125.4 (8)
O3A—C5A—H5B	109.2	C20H—C15H—C14H	118.8 (8)
C4A—C5A—H5B	109.2	C17A—C16A—C15A	121.7 (9)
H5A—C5A—H5B	107.9	C17A—C16A—H16A	119.2
O3B—C5B—C4B	112.2 (8)	C15A—C16A—H16A	119.2
O3B—C5B—H5C	109.2	C17B—C16B—C15B	121.4 (9)
C4B—C5B—H5C	109.2	C17B—C16B—H16B	119.3
O3B—C5B—H5D	109.2	C15B—C16B—H16B	119.3
C4B—C5B—H5D	109.2	C17C—C16C—C15C	121.7 (9)
H5C—C5B—H5D	107.9	C17C—C16C—H16C	119.2
O3C—C5C—C4C	113.6 (8)	C15C—C16C—H16C	119.2
O3C—C5C—H5E	108.8	C15D—C16D—C17D	121.8 (9)
C4C—C5C—H5E	108.8	C15D—C16D—H16D	119.1
O3C—C5C—H5F	108.8	C17D—C16D—H16D	119.1
C4C—C5C—H5F	108.8	C17E—C16E—C15E	120.9 (9)
H5E—C5C—H5F	107.7	C17E—C16E—H16E	119.6
O3D—C5D—C4D	113.2 (8)	C15E—C16E—H16E	119.6
O3D—C5D—H5G	108.9	C17F—C16F—C15F	122.0 (9)
C4D—C5D—H5G	108.9	C17F—C16F—H16F	119.0
O3D—C5D—H5H	108.9	C15F—C16F—H16F	119.0
C4D—C5D—H5H	108.9	C17G—C16G—C15G	122.5 (9)
H5G—C5D—H5H	107.7	C17G—C16G—H16G	118.7
O3E—C5E—C4E	112.7 (8)	C15G—C16G—H16G	118.7
O3E—C5E—H5I	109.0	C15H—C16H—C17H	121.1 (9)
C4E—C5E—H5I	109.0	C15H—C16H—H16H	119.5
O3E—C5E—H5J	109.0	C17H—C16H—H16H	119.5
C4E—C5E—H5J	109.0	C18A—C17A—C16A	121.7 (9)
H5I—C5E—H5J	107.8	C18A—C17A—H17A	119.1
O3F—C5F—C4F	112.6 (8)	C16A—C17A—H17A	119.1
O3F—C5F—H5K	109.1	C16B—C17B—C18B	121.8 (10)
C4F—C5F—H5K	109.1	C16B—C17B—H17B	119.1
O3F—C5F—H5L	109.1	C18B—C17B—H17B	119.1
C4F—C5F—H5L	109.1	C16C—C17C—C18C	121.0 (9)
H5K—C5F—H5L	107.8	C16C—C17C—H17C	119.5
O3G—C5G—C4G	112.5 (8)	C18C—C17C—H17C	119.5
O3G—C5G—H5M	109.1	C16D—C17D—C18D	120.7 (9)
C4G—C5G—H5M	109.1	C16D—C17D—H17D	119.6
O3G—C5G—H5N	109.1	C18D—C17D—H17D	119.6
C4G—C5G—H5N	109.1	C18E—C17E—C16E	122.1 (9)
H5M—C5G—H5N	107.8	C18E—C17E—H17E	118.9
O3H—C5H—C4H	112.7 (8)	C16E—C17E—H17E	118.9
O3H—C5H—H5O	109.0	C16F—C17F—C18F	120.4 (9)
C4H—C5H—H5O	109.0	C16F—C17F—H17F	119.8
O3H—C5H—H5P	109.0	C18F—C17F—H17F	119.8
C4H—C5H—H5P	109.0	C16G—C17G—C18G	120.0 (9)
H5O—C5H—H5P	107.8	C16G—C17G—H17G	120.0
O3A—C6A—C7A	117.9 (9)	C18G—C17G—H17G	120.0
O3A—C6A—C11A	120.7 (8)	C18H—C17H—C16H	121.6 (9)
C7A—C6A—C11A	121.3 (10)	C18H—C17H—H17H	119.2
O3B—C6B—C7B	118.6 (8)	C16H—C17H—H17H	119.2
O3B—C6B—C11B	121.1 (8)	C17A—C18A—C19A	117.8 (9)
C7B—C6B—C11B	120.2 (10)	C17A—C18A—C21A	122.1 (10)
O3C—C6C—C11C	120.9 (8)	C19A—C18A—C21A	120.1 (10)
O3C—C6C—C7C	117.7 (8)	C17B—C18B—C19B	117.5 (9)
C11C—C6C—C7C	121.3 (9)	C17B—C18B—C21B	123.2 (10)
O3D—C6D—C7D	118.1 (8)	C19B—C18B—C21B	119.3 (9)
O3D—C6D—C11D	120.2 (7)	C17C—C18C—C19C	118.3 (8)
C7D—C6D—C11D	121.5 (9)	C17C—C18C—C21C	121.1 (10)
O3E—C6E—C7E	117.8 (9)	C19C—C18C—C21C	120.5 (10)
O3E—C6E—C11E	122.1 (9)	C19D—C18D—C17D	117.7 (9)
C7E—C6E—C11E	120.1 (10)	C19D—C18D—C21D	121.1 (9)
O3F—C6F—C7F	117.9 (9)	C17D—C18D—C21D	121.0 (10)
O3F—C6F—C11F	120.5 (8)	C17E—C18E—C19E	118.1 (9)
C7F—C6F—C11F	121.3 (9)	C17E—C18E—C21E	122.8 (10)
O3G—C6G—C11G	120.9 (8)	C19E—C18E—C21E	119.1 (9)
O3G—C6G—C7G	117.9 (9)	C19F—C18F—C17F	117.5 (9)
C11G—C6G—C7G	121.1 (10)	C19F—C18F—C21F	122.2 (10)
O3H—C6H—C7H	118.4 (8)	C17F—C18F—C21F	120.3 (10)
O3H—C6H—C11H	120.3 (8)	C19G—C18G—C17G	118.4 (9)
C7H—C6H—C11H	121.2 (9)	C19G—C18G—C21G	120.1 (9)
C8A—C7A—C6A	120.0 (11)	C17G—C18G—C21G	121.4 (10)
C8A—C7A—H7A	120.0	C19H—C18H—C17H	117.8 (8)
C6A—C7A—H7A	120.0	C19H—C18H—C21H	121.6 (9)
C8B—C7B—C6B	120.5 (10)	C17H—C18H—C21H	120.6 (9)
C8B—C7B—H7B	119.7	C20A—C19A—C18A	120.3 (9)
C6B—C7B—H7B	119.7	C20A—C19A—H19A	119.8
C8C—C7C—C6C	120.3 (9)	C18A—C19A—H19A	119.8
C8C—C7C—H7C	119.8	C18B—C19B—C20B	120.1 (9)
C6C—C7C—H7C	119.8	C18B—C19B—H19B	119.9
C8D—C7D—C6D	119.6 (10)	C20B—C19B—H19B	119.9
C8D—C7D—H7D	120.2	C20C—C19C—C18C	120.0 (9)
C6D—C7D—H7D	120.2	C20C—C19C—H19C	120.0
C8E—C7E—C6E	119.6 (10)	C18C—C19C—H19C	120.0
C8E—C7E—H7E	120.2	C18D—C19D—C20D	120.8 (9)
C6E—C7E—H7E	120.2	C18D—C19D—H19D	119.6
C8F—C7F—C6F	120.5 (10)	C20D—C19D—H19D	119.6
C8F—C7F—H7F	119.8	C20E—C19E—C18E	119.8 (9)
C6F—C7F—H7F	119.8	C20E—C19E—H19E	120.1
C8G—C7G—C6G	119.3 (10)	C18E—C19E—H19E	120.1
C8G—C7G—H7G	120.3	C20F—C19F—C18F	121.9 (9)
C6G—C7G—H7G	120.3	C20F—C19F—H19F	119.1
C8H—C7H—C6H	121.1 (10)	C18F—C19F—H19F	119.1
C8H—C7H—H7H	119.5	C18G—C19G—C20G	120.8 (9)
C6H—C7H—H7H	119.5	C18G—C19G—H19G	119.6
C7A—C8A—C9A	120.6 (10)	C20G—C19G—H19G	119.6
C7A—C8A—H8A	119.7	C18H—C19H—C20H	121.1 (9)
C9A—C8A—H8A	119.7	C18H—C19H—H19H	119.5
C7B—C8B—C9B	120.7 (9)	C20H—C19H—H19H	119.5
C7B—C8B—H8B	119.6	C19A—C20A—O1A	115.6 (8)
C9B—C8B—H8B	119.6	C19A—C20A—C15A	123.1 (9)
C7C—C8C—C9C	119.7 (10)	O1A—C20A—C15A	121.3 (8)
C7C—C8C—H8C	120.1	O1B—C20B—C15B	122.2 (9)
C9C—C8C—H8C	120.2	O1B—C20B—C19B	115.3 (8)
C7D—C8D—C9D	121.2 (10)	C15B—C20B—C19B	122.6 (9)
C7D—C8D—H8D	119.4	O1C—C20C—C19C	115.3 (9)
C9D—C8D—H8D	119.4	O1C—C20C—C15C	122.5 (8)
C7E—C8E—C9E	121.2 (10)	C19C—C20C—C15C	122.2 (9)
C7E—C8E—H8E	119.4	C19D—C20D—O1D	115.7 (8)
C9E—C8E—H8E	119.4	C19D—C20D—C15D	122.9 (9)
C7F—C8F—C9F	120.3 (10)	O1D—C20D—C15D	121.4 (8)
C7F—C8F—H8F	119.8	C15E—C20E—C19E	122.8 (9)
C9F—C8F—H8F	119.8	C15E—C20E—O1E	121.6 (8)
C7G—C8G—C9G	121.6 (10)	C19E—C20E—O1E	115.6 (8)
C7G—C8G—H8G	119.2	C19F—C20F—O1F	116.0 (8)
C9G—C8G—H8G	119.2	C19F—C20F—C15F	122.3 (9)
C7H—C8H—C9H	119.8 (10)	O1F—C20F—C15F	121.6 (8)
C7H—C8H—H8H	120.1	O1G—C20G—C19G	115.9 (8)
C9H—C8H—H8H	120.1	O1G—C20G—C15G	122.1 (8)
C8A—C9A—C10A	120.1 (10)	C19G—C20G—C15G	122.1 (9)
C8A—C9A—H9A	119.9	C19H—C20H—O1H	115.8 (8)
C10A—C9A—H9A	119.9	C19H—C20H—C15H	122.7 (9)
C8B—C9B—C10B	119.7 (10)	O1H—C20H—C15H	121.5 (8)
C8B—C9B—H9B	120.2	C18A—C21A—H21A	109.5
C10B—C9B—H9B	120.2	C18A—C21A—H21B	109.5
C8C—C9C—C10C	119.3 (10)	H21A—C21A—H21B	109.5
C8C—C9C—H9C	120.3	C18A—C21A—H21C	109.5
C10C—C9C—H9C	120.3	H21A—C21A—H21C	109.5
C8D—C9D—C10D	119.2 (10)	H21B—C21A—H21C	109.5
C8D—C9D—H9D	120.4	C18B—C21B—H21D	109.5
C10D—C9D—H9D	120.4	C18B—C21B—H21E	109.5
C8E—C9E—C10E	120.1 (10)	H21D—C21B—H21E	109.5
C8E—C9E—H9E	120.0	C18B—C21B—H21F	109.5
C10E—C9E—H9E	120.0	H21D—C21B—H21F	109.5
C8F—C9F—C10F	120.1 (10)	H21E—C21B—H21F	109.5
C8F—C9F—H9F	119.9	C18C—C21C—H21G	109.5
C10F—C9F—H9F	119.9	C18C—C21C—H21H	109.5
C8G—C9G—C10G	119.1 (10)	H21G—C21C—H21H	109.5
C8G—C9G—H9G	120.5	C18C—C21C—H21I	109.5
C10G—C9G—H9G	120.4	H21G—C21C—H21I	109.5
C8H—C9H—C10H	119.3 (10)	H21H—C21C—H21I	109.5
C8H—C9H—H9H	120.3	C18D—C21D—H21J	109.5
C10H—C9H—H9H	120.3	C18D—C21D—H21K	109.5
C11A—C10A—C9A	120.6 (10)	H21J—C21D—H21K	109.5
C11A—C10A—H10A	119.7	C18D—C21D—H21L	109.5
C9A—C10A—H10A	119.7	H21J—C21D—H21L	109.5
C9B—C10B—C11B	121.5 (9)	H21K—C21D—H21L	109.5
C9B—C10B—H10B	119.2	C18E—C21E—H21M	109.5
C11B—C10B—H10B	119.2	C18E—C21E—H21N	109.5
C11C—C10C—C9C	122.2 (9)	H21M—C21E—H21N	109.5
C11C—C10C—H10C	118.9	C18E—C21E—H21O	109.5
C9C—C10C—H10C	118.9	H21M—C21E—H21O	109.5
C11D—C10D—C9D	119.8 (9)	H21N—C21E—H21O	109.5
C11D—C10D—H10D	120.1	C18F—C21F—H21P	109.5
C9D—C10D—H10D	120.1	C18F—C21F—H21Q	109.5
C11E—C10E—C9E	120.1 (10)	H21P—C21F—H21Q	109.5
C11E—C10E—H10E	119.9	C18F—C21F—H21R	109.5
C9E—C10E—H10E	119.9	H21P—C21F—H21R	109.5
C11F—C10F—C9F	120.1 (10)	H21Q—C21F—H21R	109.5
C11F—C10F—H10F	119.9	C18G—C21G—H21S	109.5
C9F—C10F—H10F	119.9	C18G—C21G—H21T	109.5
C9G—C10G—C11G	121.3 (9)	H21S—C21G—H21T	109.5
C9G—C10G—H10G	119.3	C18G—C21G—H21U	109.5
C11G—C10G—H10G	119.3	H21S—C21G—H21U	109.5
C11H—C10H—C9H	121.6 (9)	H21T—C21G—H21U	109.5
C11H—C10H—H10H	119.2	C18H—C21H—H21V	109.5
C9H—C10H—H10H	119.2	C18H—C21H—H21W	109.5
C10A—C11A—C6A	117.3 (9)	H21V—C21H—H21W	109.5
C10A—C11A—C12A	124.8 (8)	C18H—C21H—H21X	109.5
C6A—C11A—C12A	118.0 (8)	H21V—C21H—H21X	109.5
C10B—C11B—C6B	117.3 (8)	H21W—C21H—H21X	109.5
C10B—C11B—C12B	125.4 (8)	C1A—O1A—C20A	122.1 (7)
C6B—C11B—C12B	117.3 (8)	C1B—O1B—C20B	121.7 (7)
C10C—C11C—C6C	117.0 (9)	C1C—O1C—C20C	121.5 (7)
C10C—C11C—C12C	125.1 (8)	C1D—O1D—C20D	122.4 (7)
C6C—C11C—C12C	117.9 (8)	C1E—O1E—C20E	121.3 (7)
C6D—C11D—C10D	118.4 (8)	C1F—O1F—C20F	122.5 (7)
C6D—C11D—C12D	119.0 (8)	C1G—O1G—C20G	121.0 (7)
C10D—C11D—C12D	122.5 (8)	C1H—O1H—C20H	122.0 (7)
C10E—C11E—C6E	118.8 (9)	C6A—O3A—C5A	113.7 (7)
C10E—C11E—C12E	124.0 (8)	C6B—O3B—C5B	114.0 (7)
C6E—C11E—C12E	117.2 (8)	C6C—O3C—C5C	113.2 (7)
C10F—C11F—C6F	117.5 (8)	C6D—O3D—C5D	113.6 (7)
C10F—C11F—C12F	124.1 (8)	C6E—O3E—C5E	114.2 (7)
C6F—C11F—C12F	118.5 (8)	C6F—O3F—C5F	113.6 (7)
C6G—C11G—C10G	117.5 (8)	C6G—O3G—C5G	114.1 (7)
C6G—C11G—C12G	118.6 (8)	C6H—O3H—C5H	113.6 (7)
			
O2A—C1A—C2A—C14A	179.6 (9)	C4G—C12G—C13G—C14G	−0.9 (12)
O1A—C1A—C2A—C14A	−1.7 (13)	C11G—C12G—C13G—C14G	−180.0 (7)
O2A—C1A—C2A—C3A	0.0 (15)	C4H—C12H—C13H—C14H	0.2 (12)
O1A—C1A—C2A—C3A	178.6 (8)	C11H—C12H—C13H—C14H	179.5 (8)
O2B—C1B—C2B—C14B	179.7 (9)	C3A—C2A—C14A—C13A	2.1 (12)
O1B—C1B—C2B—C14B	0.2 (13)	C1A—C2A—C14A—C13A	−177.5 (8)
O2B—C1B—C2B—C3B	−0.8 (14)	C3A—C2A—C14A—C15A	−179.0 (8)
O1B—C1B—C2B—C3B	179.7 (8)	C1A—C2A—C14A—C15A	1.3 (12)
O2C—C1C—C2C—C3C	−0.6 (15)	C12A—C13A—C14A—C2A	−1.0 (12)
O1C—C1C—C2C—C3C	−179.9 (8)	C12A—C13A—C14A—C15A	−179.8 (7)
O2C—C1C—C2C—C14C	−179.6 (9)	C3B—C2B—C14B—C13B	1.9 (12)
O1C—C1C—C2C—C14C	1.1 (13)	C1B—C2B—C14B—C13B	−178.6 (8)
O2D—C1D—C2D—C14D	180.0 (9)	C3B—C2B—C14B—C15B	−179.1 (8)
O1D—C1D—C2D—C14D	0.4 (13)	C1B—C2B—C14B—C15B	0.4 (12)
O2D—C1D—C2D—C3D	0.4 (15)	C12B—C13B—C14B—C2B	−0.4 (12)
O1D—C1D—C2D—C3D	−179.2 (8)	C12B—C13B—C14B—C15B	−179.4 (7)
O2E—C1E—C2E—C14E	−179.2 (9)	C3C—C2C—C14C—C13C	−1.3 (13)
O1E—C1E—C2E—C14E	1.3 (13)	C1C—C2C—C14C—C13C	177.6 (8)
O2E—C1E—C2E—C3E	−0.9 (14)	C3C—C2C—C14C—C15C	179.7 (8)
O1E—C1E—C2E—C3E	179.5 (8)	C1C—C2C—C14C—C15C	−1.3 (12)
O2F—C1F—C2F—C14F	−178.2 (9)	C12C—C13C—C14C—C2C	−0.3 (12)
O1F—C1F—C2F—C14F	0.2 (13)	C12C—C13C—C14C—C15C	178.7 (7)
O2F—C1F—C2F—C3F	2.6 (15)	C3D—C2D—C14D—C13D	−3.2 (12)
O1F—C1F—C2F—C3F	−179.0 (8)	C1D—C2D—C14D—C13D	177.2 (8)
O2G—C1G—C2G—C14G	179.5 (9)	C3D—C2D—C14D—C15D	179.7 (8)
O1G—C1G—C2G—C14G	1.6 (13)	C1D—C2D—C14D—C15D	0.1 (12)
O2G—C1G—C2G—C3G	−2.1 (14)	C12D—C13D—C14D—C2D	2.7 (12)
O1G—C1G—C2G—C3G	180.0 (8)	C12D—C13D—C14D—C15D	179.7 (7)
O2H—C1H—C2H—C14H	180.0 (9)	C3E—C2E—C14E—C13E	−0.9 (12)
O1H—C1H—C2H—C14H	−1.4 (13)	C1E—C2E—C14E—C13E	177.3 (8)
O2H—C1H—C2H—C3H	0.6 (15)	C3E—C2E—C14E—C15E	−179.7 (7)
O1H—C1H—C2H—C3H	179.2 (8)	C1E—C2E—C14E—C15E	−1.5 (12)
C14A—C2A—C3A—C4A	−1.9 (13)	C12E—C13E—C14E—C2E	0.5 (12)
C1A—C2A—C3A—C4A	177.7 (8)	C12E—C13E—C14E—C15E	179.2 (7)
C14B—C2B—C3B—C4B	−2.6 (13)	C3F—C2F—C14F—C13F	−2.6 (12)
C1B—C2B—C3B—C4B	177.9 (8)	C1F—C2F—C14F—C13F	178.2 (8)
C14C—C2C—C3C—C4C	1.3 (14)	C3F—C2F—C14F—C15F	179.1 (8)
C1C—C2C—C3C—C4C	−177.8 (8)	C1F—C2F—C14F—C15F	−0.1 (12)
C14D—C2D—C3D—C4D	2.1 (13)	C12F—C13F—C14F—C2F	1.6 (12)
C1D—C2D—C3D—C4D	−178.2 (8)	C12F—C13F—C14F—C15F	179.8 (8)
C14E—C2E—C3E—C4E	1.0 (13)	C3G—C2G—C14G—C13G	3.2 (12)
C1E—C2E—C3E—C4E	−177.2 (8)	C1G—C2G—C14G—C13G	−178.4 (8)
C14F—C2F—C3F—C4F	2.7 (13)	C3G—C2G—C14G—C15G	−179.2 (7)
C1F—C2F—C3F—C4F	−178.1 (8)	C1G—C2G—C14G—C15G	−0.8 (12)
C14G—C2G—C3G—C4G	−3.3 (13)	C12G—C13G—C14G—C2G	−1.1 (12)
C1G—C2G—C3G—C4G	178.2 (8)	C12G—C13G—C14G—C15G	−178.5 (7)
C14H—C2H—C3H—C4H	−2.1 (13)	C3H—C2H—C14H—C13H	2.0 (12)
C1H—C2H—C3H—C4H	177.4 (8)	C1H—C2H—C14H—C13H	−177.4 (8)
C2A—C3A—C4A—C12A	0.4 (14)	C3H—C2H—C14H—C15H	−179.6 (8)
C2A—C3A—C4A—C5A	−178.1 (8)	C1H—C2H—C14H—C15H	1.0 (12)
C2B—C3B—C4B—C12B	1.8 (13)	C12H—C13H—C14H—C2H	−1.1 (12)
C2B—C3B—C4B—C5B	−177.5 (8)	C12H—C13H—C14H—C15H	−179.4 (7)
C2C—C3C—C4C—C12C	0.5 (13)	C2A—C14A—C15A—C16A	177.6 (8)
C2C—C3C—C4C—C5C	177.4 (9)	C13A—C14A—C15A—C16A	−3.6 (13)
C2D—C3D—C4D—C12D	−0.6 (13)	C2A—C14A—C15A—C20A	−0.6 (12)
C2D—C3D—C4D—C5D	178.0 (8)	C13A—C14A—C15A—C20A	178.2 (7)
C2E—C3E—C4E—C12E	−0.6 (13)	C2B—C14B—C15B—C20B	−1.0 (12)
C2E—C3E—C4E—C5E	178.7 (8)	C13B—C14B—C15B—C20B	177.9 (8)
C2F—C3F—C4F—C12F	−1.7 (13)	C2B—C14B—C15B—C16B	178.1 (8)
C2F—C3F—C4F—C5F	178.8 (8)	C13B—C14B—C15B—C16B	−3.0 (13)
C2G—C3G—C4G—C12G	1.3 (13)	C2C—C14C—C15C—C20C	1.1 (11)
C2G—C3G—C4G—C5G	−176.7 (8)	C13C—C14C—C15C—C20C	−177.8 (8)
C2H—C3H—C4H—C12H	1.2 (14)	C2C—C14C—C15C—C16C	−177.9 (8)
C2H—C3H—C4H—C5H	−178.7 (8)	C13C—C14C—C15C—C16C	3.2 (13)
C3A—C4A—C5A—O3A	−147.0 (9)	C2D—C14D—C15D—C16D	−178.0 (8)
C12A—C4A—C5A—O3A	34.4 (11)	C13D—C14D—C15D—C16D	5.0 (13)
C12B—C4B—C5B—O3B	33.2 (11)	C2D—C14D—C15D—C20D	−0.5 (12)
C3B—C4B—C5B—O3B	−147.5 (8)	C13D—C14D—C15D—C20D	−177.5 (8)
C3C—C4C—C5C—O3C	147.5 (9)	C2E—C14E—C15E—C20E	0.4 (11)
C12C—C4C—C5C—O3C	−35.5 (12)	C13E—C14E—C15E—C20E	−178.3 (8)
C3D—C4D—C5D—O3D	147.2 (9)	C2E—C14E—C15E—C16E	−177.4 (8)
C12D—C4D—C5D—O3D	−34.1 (12)	C13E—C14E—C15E—C16E	3.9 (13)
C3E—C4E—C5E—O3E	147.7 (8)	C2F—C14F—C15F—C20F	0.2 (12)
C12E—C4E—C5E—O3E	−32.9 (12)	C13F—C14F—C15F—C20F	−178.1 (8)
C3F—C4F—C5F—O3F	146.9 (9)	C2F—C14F—C15F—C16F	−177.7 (8)
C12F—C4F—C5F—O3F	−32.7 (12)	C13F—C14F—C15F—C16F	4.0 (13)
C3G—C4G—C5G—O3G	−147.4 (8)	C2G—C14G—C15G—C20G	0.2 (11)
C12G—C4G—C5G—O3G	34.6 (12)	C13G—C14G—C15G—C20G	177.7 (7)
C3H—C4H—C5H—O3H	−147.1 (9)	C2G—C14G—C15G—C16G	178.2 (8)
C12H—C4H—C5H—O3H	32.9 (12)	C13G—C14G—C15G—C16G	−4.3 (13)
O3A—C6A—C7A—C8A	−179.7 (9)	C2H—C14H—C15H—C16H	178.4 (8)
C11A—C6A—C7A—C8A	−3.8 (16)	C13H—C14H—C15H—C16H	−3.2 (14)
O3B—C6B—C7B—C8B	−179.0 (9)	C2H—C14H—C15H—C20H	0.1 (12)
C11B—C6B—C7B—C8B	−1.5 (15)	C13H—C14H—C15H—C20H	178.4 (8)
O3C—C6C—C7C—C8C	178.6 (9)	C20A—C15A—C16A—C17A	−1.1 (13)
C11C—C6C—C7C—C8C	1.8 (15)	C14A—C15A—C16A—C17A	−179.2 (8)
O3D—C6D—C7D—C8D	177.5 (9)	C20B—C15B—C16B—C17B	0.1 (13)
C11D—C6D—C7D—C8D	0.7 (15)	C14B—C15B—C16B—C17B	−179.0 (8)
O3E—C6E—C7E—C8E	179.9 (10)	C20C—C15C—C16C—C17C	0.0 (13)
C11E—C6E—C7E—C8E	2.7 (15)	C14C—C15C—C16C—C17C	179.0 (8)
O3F—C6F—C7F—C8F	177.3 (9)	C20D—C15D—C16D—C17D	0.9 (13)
C11F—C6F—C7F—C8F	3.6 (16)	C14D—C15D—C16D—C17D	178.5 (8)
O3G—C6G—C7G—C8G	179.9 (10)	C20E—C15E—C16E—C17E	0.9 (12)
C11G—C6G—C7G—C8G	−3.2 (16)	C14E—C15E—C16E—C17E	178.7 (8)
O3H—C6H—C7H—C8H	−176.8 (10)	C20F—C15F—C16F—C17F	1.0 (13)
C11H—C6H—C7H—C8H	−1.5 (16)	C14F—C15F—C16F—C17F	178.9 (9)
C6A—C7A—C8A—C9A	1.9 (17)	C20G—C15G—C16G—C17G	−0.9 (13)
C6B—C7B—C8B—C9B	−0.2 (17)	C14G—C15G—C16G—C17G	−178.9 (8)
C6C—C7C—C8C—C9C	1.5 (16)	C20H—C15H—C16H—C17H	−1.0 (13)
C6D—C7D—C8D—C9D	1.4 (16)	C14H—C15H—C16H—C17H	−179.4 (8)
C6E—C7E—C8E—C9E	0.1 (17)	C15A—C16A—C17A—C18A	−0.2 (15)
C6F—C7F—C8F—C9F	−0.4 (17)	C15B—C16B—C17B—C18B	−1.9 (15)
C6G—C7G—C8G—C9G	1.0 (17)	C15C—C16C—C17C—C18C	1.8 (14)
C6H—C7H—C8H—C9H	−0.9 (17)	C15D—C16D—C17D—C18D	1.1 (15)
C7A—C8A—C9A—C10A	0.6 (16)	C15E—C16E—C17E—C18E	1.4 (15)
C7B—C8B—C9B—C10B	1.9 (16)	C15F—C16F—C17F—C18F	−0.1 (15)
C7C—C8C—C9C—C10C	−3.3 (16)	C15G—C16G—C17G—C18G	−1.1 (14)
C7D—C8D—C9D—C10D	−1.3 (16)	C15H—C16H—C17H—C18H	0.1 (15)
C7E—C8E—C9E—C10E	−1.7 (17)	C16A—C17A—C18A—C19A	1.0 (15)
C7F—C8F—C9F—C10F	−1.2 (16)	C16A—C17A—C18A—C21A	179.1 (9)
C7G—C8G—C9G—C10G	1.6 (17)	C16B—C17B—C18B—C19B	2.0 (15)
C7H—C8H—C9H—C10H	2.0 (17)	C16B—C17B—C18B—C21B	179.1 (9)
C8A—C9A—C10A—C11A	−1.3 (15)	C16C—C17C—C18C—C19C	−1.9 (13)
C8B—C9B—C10B—C11B	−1.9 (15)	C16C—C17C—C18C—C21C	−179.7 (8)
C8C—C9C—C10C—C11C	1.9 (15)	C16D—C17D—C18D—C19D	−2.5 (14)
C8D—C9D—C10D—C11D	−1.0 (14)	C16D—C17D—C18D—C21D	−178.2 (9)
C8E—C9E—C10E—C11E	0.6 (15)	C16E—C17E—C18E—C19E	−2.1 (14)
C8F—C9F—C10F—C11F	−0.5 (15)	C16E—C17E—C18E—C21E	179.4 (9)
C8G—C9G—C10G—C11G	−1.9 (15)	C16F—C17F—C18F—C19F	−0.5 (14)
C8H—C9H—C10H—C11H	−0.7 (15)	C16F—C17F—C18F—C21F	−178.7 (9)
C9A—C10A—C11A—C6A	−0.6 (13)	C16G—C17G—C18G—C19G	2.5 (14)
C9A—C10A—C11A—C12A	178.6 (9)	C16G—C17G—C18G—C21G	178.8 (8)
O3A—C6A—C11A—C10A	178.9 (8)	C16H—C17H—C18H—C19H	0.4 (14)
C7A—C6A—C11A—C10A	3.1 (14)	C16H—C17H—C18H—C21H	178.4 (9)
O3A—C6A—C11A—C12A	−0.3 (13)	C17A—C18A—C19A—C20A	−0.5 (14)
C7A—C6A—C11A—C12A	−176.1 (9)	C21A—C18A—C19A—C20A	−178.7 (9)
C9B—C10B—C11B—C6B	0.2 (13)	C17B—C18B—C19B—C20B	−0.3 (14)
C9B—C10B—C11B—C12B	178.2 (9)	C21B—C18B—C19B—C20B	−177.6 (9)
O3B—C6B—C11B—C10B	178.9 (8)	C17C—C18C—C19C—C20C	0.1 (13)
C7B—C6B—C11B—C10B	1.4 (13)	C21C—C18C—C19C—C20C	178.0 (8)
O3B—C6B—C11B—C12B	0.7 (12)	C17D—C18D—C19D—C20D	1.8 (14)
C7B—C6B—C11B—C12B	−176.7 (8)	C21D—C18D—C19D—C20D	177.5 (9)
C9C—C10C—C11C—C6C	1.3 (13)	C17E—C18E—C19E—C20E	0.5 (13)
C9C—C10C—C11C—C12C	−179.7 (9)	C21E—C18E—C19E—C20E	179.1 (9)
O3C—C6C—C11C—C10C	−179.8 (8)	C17F—C18F—C19F—C20F	0.3 (14)
C7C—C6C—C11C—C10C	−3.1 (13)	C21F—C18F—C19F—C20F	178.4 (9)
O3C—C6C—C11C—C12C	1.1 (12)	C17G—C18G—C19G—C20G	−1.9 (14)
C7C—C6C—C11C—C12C	177.8 (8)	C21G—C18G—C19G—C20G	−178.2 (8)
O3D—C6D—C11D—C10D	−179.6 (8)	C17H—C18H—C19H—C20H	0.0 (14)
C7D—C6D—C11D—C10D	−2.9 (13)	C21H—C18H—C19H—C20H	−178.0 (9)
O3D—C6D—C11D—C12D	0.9 (12)	C18A—C19A—C20A—O1A	179.0 (8)
C7D—C6D—C11D—C12D	177.6 (8)	C18A—C19A—C20A—C15A	−0.8 (14)
C9D—C10D—C11D—C6D	3.0 (13)	C16A—C15A—C20A—C19A	1.5 (13)
C9D—C10D—C11D—C12D	−177.5 (9)	C14A—C15A—C20A—C19A	179.8 (8)
C9E—C10E—C11E—C6E	2.1 (13)	C16A—C15A—C20A—O1A	−178.2 (8)
C9E—C10E—C11E—C12E	−178.2 (9)	C14A—C15A—C20A—O1A	0.1 (12)
O3E—C6E—C11E—C10E	179.2 (8)	C16B—C15B—C20B—O1B	−178.1 (8)
C7E—C6E—C11E—C10E	−3.7 (13)	C14B—C15B—C20B—O1B	1.0 (12)
O3E—C6E—C11E—C12E	−0.6 (13)	C16B—C15B—C20B—C19B	1.6 (13)
C7E—C6E—C11E—C12E	176.5 (8)	C14B—C15B—C20B—C19B	−179.2 (8)
C9F—C10F—C11F—C6F	3.5 (13)	C18B—C19B—C20B—O1B	178.2 (8)
C9F—C10F—C11F—C12F	−178.0 (9)	C18B—C19B—C20B—C15B	−1.5 (14)
O3F—C6F—C11F—C10F	−178.6 (8)	C18C—C19C—C20C—O1C	−178.4 (8)
C7F—C6F—C11F—C10F	−5.0 (13)	C18C—C19C—C20C—C15C	1.8 (13)
O3F—C6F—C11F—C12F	2.7 (12)	C16C—C15C—C20C—O1C	178.4 (8)
C7F—C6F—C11F—C12F	176.3 (9)	C14C—C15C—C20C—O1C	−0.7 (12)
O3G—C6G—C11G—C10G	179.5 (8)	C16C—C15C—C20C—C19C	−1.8 (13)
C7G—C6G—C11G—C10G	2.8 (14)	C14C—C15C—C20C—C19C	179.1 (8)
O3G—C6G—C11G—C12G	0.4 (13)	C18D—C19D—C20D—O1D	−179.5 (8)
C7G—C6G—C11G—C12G	−176.4 (9)	C18D—C19D—C20D—C15D	0.3 (14)
C9G—C10G—C11G—C6G	−0.2 (13)	C16D—C15D—C20D—C19D	−1.7 (13)
C9G—C10G—C11G—C12G	178.9 (8)	C14D—C15D—C20D—C19D	−179.4 (8)
C9H—C10H—C11H—C6H	−1.6 (13)	C16D—C15D—C20D—O1D	178.2 (8)
C9H—C10H—C11H—C12H	177.9 (9)	C14D—C15D—C20D—O1D	0.4 (13)
O3H—C6H—C11H—C10H	178.0 (8)	C16E—C15E—C20E—C19E	−2.5 (12)
C7H—C6H—C11H—C10H	2.7 (13)	C14E—C15E—C20E—C19E	179.5 (8)
O3H—C6H—C11H—C12H	−1.5 (13)	C16E—C15E—C20E—O1E	178.9 (8)
C7H—C6H—C11H—C12H	−176.8 (9)	C14E—C15E—C20E—O1E	0.9 (12)
C3A—C4A—C12A—C13A	0.7 (13)	C18E—C19E—C20E—C15E	1.9 (13)
C5A—C4A—C12A—C13A	179.3 (8)	C18E—C19E—C20E—O1E	−179.4 (8)
C3A—C4A—C12A—C11A	−179.5 (8)	C18F—C19F—C20F—O1F	−178.5 (8)
C5A—C4A—C12A—C11A	−0.9 (12)	C18F—C19F—C20F—C15F	0.6 (14)
C10A—C11A—C12A—C4A	163.7 (8)	C16F—C15F—C20F—C19F	−1.2 (13)
C6A—C11A—C12A—C4A	−17.1 (12)	C14F—C15F—C20F—C19F	−179.3 (8)
C10A—C11A—C12A—C13A	−16.5 (13)	C16F—C15F—C20F—O1F	177.8 (8)
C6A—C11A—C12A—C13A	162.7 (8)	C14F—C15F—C20F—O1F	−0.3 (13)
C3B—C4B—C12B—C13B	−0.4 (12)	C18G—C19G—C20G—O1G	179.8 (8)
C5B—C4B—C12B—C13B	178.9 (8)	C18G—C19G—C20G—C15G	−0.2 (14)
C3B—C4B—C12B—C11B	−178.9 (8)	C16G—C15G—C20G—O1G	−178.4 (8)
C5B—C4B—C12B—C11B	0.4 (11)	C14G—C15G—C20G—O1G	−0.3 (12)
C10B—C11B—C12B—C4B	163.8 (8)	C16G—C15G—C20G—C19G	1.6 (12)
C6B—C11B—C12B—C4B	−18.3 (11)	C14G—C15G—C20G—C19G	179.7 (8)
C10B—C11B—C12B—C13B	−14.7 (13)	C18H—C19H—C20H—O1H	179.7 (8)
C6B—C11B—C12B—C13B	163.3 (8)	C18H—C19H—C20H—C15H	−0.9 (14)
C3C—C4C—C12C—C13C	−2.1 (12)	C16H—C15H—C20H—C19H	1.4 (13)
C5C—C4C—C12C—C13C	−179.1 (8)	C14H—C15H—C20H—C19H	179.9 (8)
C3C—C4C—C12C—C11C	179.5 (8)	C16H—C15H—C20H—O1H	−179.3 (8)
C5C—C4C—C12C—C11C	2.4 (12)	C14H—C15H—C20H—O1H	−0.8 (12)
C10C—C11C—C12C—C4C	−163.9 (8)	O2A—C1A—O1A—C20A	−179.9 (8)
C6C—C11C—C12C—C4C	15.1 (11)	C2A—C1A—O1A—C20A	1.3 (13)
C10C—C11C—C12C—C13C	17.8 (13)	C19A—C20A—O1A—C1A	179.7 (8)
C6C—C11C—C12C—C13C	−163.2 (8)	C15A—C20A—O1A—C1A	−0.6 (13)
C3D—C4D—C12D—C13D	0.1 (13)	O2B—C1B—O1B—C20B	−179.8 (8)
C5D—C4D—C12D—C13D	−178.5 (8)	C2B—C1B—O1B—C20B	−0.2 (12)
C3D—C4D—C12D—C11D	179.3 (8)	C15B—C20B—O1B—C1B	−0.4 (13)
C5D—C4D—C12D—C11D	0.7 (12)	C19B—C20B—O1B—C1B	179.8 (8)
C6D—C11D—C12D—C13D	−163.9 (8)	O2C—C1C—O1C—C20C	−180.0 (8)
C10D—C11D—C12D—C13D	16.6 (13)	C2C—C1C—O1C—C20C	−0.6 (13)
C6D—C11D—C12D—C4D	17.0 (12)	C19C—C20C—O1C—C1C	−179.4 (8)
C10D—C11D—C12D—C4D	−162.5 (8)	C15C—C20C—O1C—C1C	0.5 (13)
C3E—C4E—C12E—C13E	0.2 (12)	O2D—C1D—O1D—C20D	179.9 (8)
C5E—C4E—C12E—C13E	−179.1 (8)	C2D—C1D—O1D—C20D	−0.6 (13)
C3E—C4E—C12E—C11E	179.6 (8)	C19D—C20D—O1D—C1D	180.0 (8)
C5E—C4E—C12E—C11E	0.3 (12)	C15D—C20D—O1D—C1D	0.1 (13)
C10E—C11E—C12E—C13E	16.8 (13)	O2E—C1E—O1E—C20E	−179.5 (8)
C6E—C11E—C12E—C13E	−163.4 (8)	C2E—C1E—O1E—C20E	0.1 (12)
C10E—C11E—C12E—C4E	−162.6 (8)	C15E—C20E—O1E—C1E	−1.1 (12)
C6E—C11E—C12E—C4E	17.2 (12)	C19E—C20E—O1E—C1E	−179.9 (8)
C3F—C4F—C12F—C13F	0.6 (13)	O2F—C1F—O1F—C20F	178.2 (8)
C5F—C4F—C12F—C13F	−179.8 (8)	C2F—C1F—O1F—C20F	−0.3 (13)
C3F—C4F—C12F—C11F	179.8 (8)	C19F—C20F—O1F—C1F	179.4 (8)
C5F—C4F—C12F—C11F	−0.7 (12)	C15F—C20F—O1F—C1F	0.3 (13)
C10F—C11F—C12F—C13F	17.2 (13)	O2G—C1G—O1G—C20G	−179.7 (8)
C6F—C11F—C12F—C13F	−164.2 (8)	C2G—C1G—O1G—C20G	−1.7 (13)
C10F—C11F—C12F—C4F	−161.9 (8)	C19G—C20G—O1G—C1G	−179.0 (8)
C6F—C11F—C12F—C4F	16.7 (12)	C15G—C20G—O1G—C1G	1.0 (13)
C3G—C4G—C12G—C13G	0.8 (13)	O2H—C1H—O1H—C20H	179.4 (8)
C5G—C4G—C12G—C13G	178.9 (8)	C2H—C1H—O1H—C20H	0.6 (13)
C3G—C4G—C12G—C11G	179.9 (8)	C19H—C20H—O1H—C1H	179.8 (8)
C5G—C4G—C12G—C11G	−2.0 (12)	C15H—C20H—O1H—C1H	0.4 (13)
C6G—C11G—C12G—C13G	162.5 (8)	C7A—C6A—O3A—C5A	−149.0 (9)
C10G—C11G—C12G—C13G	−16.6 (13)	C11A—C6A—O3A—C5A	35.1 (12)
C6G—C11G—C12G—C4G	−16.6 (12)	C4A—C5A—O3A—C6A	−51.1 (10)
C10G—C11G—C12G—C4G	164.3 (8)	C7B—C6B—O3B—C5B	−148.4 (9)
C3H—C4H—C12H—C13H	−0.3 (13)	C11B—C6B—O3B—C5B	34.1 (12)
C5H—C4H—C12H—C13H	179.6 (8)	C4B—C5B—O3B—C6B	−49.9 (11)
C3H—C4H—C12H—C11H	−179.6 (8)	C11C—C6C—O3C—C5C	−34.5 (12)
C5H—C4H—C12H—C11H	0.3 (12)	C7C—C6C—O3C—C5C	148.6 (9)
C10H—C11H—C12H—C4H	163.3 (8)	C4C—C5C—O3C—C6C	51.0 (11)
C6H—C11H—C12H—C4H	−17.3 (12)	C7D—C6D—O3D—C5D	147.7 (9)
C10H—C11H—C12H—C13H	−16.0 (13)	C11D—C6D—O3D—C5D	−35.5 (12)
C6H—C11H—C12H—C13H	163.5 (8)	C4D—C5D—O3D—C6D	51.7 (11)
C4A—C12A—C13A—C14A	−0.4 (12)	C7E—C6E—O3E—C5E	149.5 (9)
C11A—C12A—C13A—C14A	179.8 (7)	C11E—C6E—O3E—C5E	−33.3 (12)
C4B—C12B—C13B—C14B	−0.4 (12)	C4E—C5E—O3E—C6E	48.9 (11)
C11B—C12B—C13B—C14B	178.1 (7)	C7F—C6F—O3F—C5F	148.7 (9)
C4C—C12C—C13C—C14C	2.0 (12)	C11F—C6F—O3F—C5F	−37.5 (11)
C11C—C12C—C13C—C14C	−179.7 (7)	C4F—C5F—O3F—C6F	51.5 (10)
C4D—C12D—C13D—C14D	−1.2 (12)	C11G—C6G—O3G—C5G	33.6 (12)
C11D—C12D—C13D—C14D	179.7 (7)	C7G—C6G—O3G—C5G	−149.5 (10)
C4E—C12E—C13E—C14E	−0.2 (12)	C4G—C5G—O3G—C6G	−50.3 (11)
C11E—C12E—C13E—C14E	−179.5 (8)	C7H—C6H—O3H—C5H	−148.5 (9)
C4F—C12F—C13F—C14F	−0.6 (13)	C11H—C6H—O3H—C5H	36.1 (12)
C11F—C12F—C13F—C14F	−179.7 (7)	C4H—C5H—O3H—C6H	−51.2 (11)

### Hydrogen-bond geometry (Å, º)

Cg24, Cg9, Cg39, Cg34, Cg4 and Cg14 are the centroids of rings C6E–C11E, C6B–C11B, C6H–C11H, C6G–C11G, C6A–C11A and C6C–C11C, respectively.

**Table:**

*D*—H···*A*	*D*—H	H···*A*	*D*···*A*	*D*—H···*A*
C7*A*—H7*A*···O3*F*^i^	0.93	2.64	3.520 (13)	157
C7*F*—H7*F*···O3*A*^ii^	0.93	2.64	3.526 (12)	159
C21*B*—H21*E*···*Cg*24	0.96	2.68	3.575 (13)	155
C21*C*—H21*H*···*Cg*9^ii^	0.96	2.80	3.554 (12)	136
C21*D*—H21*K*···*Cg*39^iii^	0.96	2.66	3.553 (12)	154
C21*E*—H21*M*···*Cg*34	0.96	2.92	3.622 (13)	131
C21*F*—H21*Q*···*Cg*4^i^	0.96	2.68	3.575 (13)	156
C21*G*—H21*S*···*Cg*14^iii^	0.96	2.66	3.556 (12)	155

Symmetry codes: (i) *x*, −*y*+1, *z*+1/2; (ii) *x*, −*y*+1, *z*−1/2; (iii) *x*+1/2, −*y*+1/2, *z*+1/2.
